# Screening for potential endocrine disruptors in fish: evidence from structural alerts and in vitro and in vivo toxicological assays

**DOI:** 10.1186/s12302-016-0094-5

**Published:** 2016-11-02

**Authors:** Monika Nendza, Andrea Wenzel, Martin Müller, Geertje Lewin, Nelly Simetska, Frauke Stock, Jürgen Arning

**Affiliations:** 1 Analytical Laboratory, Bahnhofstr. 1, 24816 Luhnstedt, Germany; 2 Fraunhofer Institute for Molecular Biology and Applied Ecology IME, Auf dem Aberg 1, 57392 Schmallenberg, Germany; 3 Fraunhofer Institute for Toxicology and Experimental Medicine ITEM, Nikolai-Fuchs-Str. 1, 30625 Hannover, Germany; 4 German Environment Agency UBA, Wörlitzer Platz 1, 06844 Dessau-Roßlau, Germany; 5 30161 Hannover, Germany

**Keywords:** REACH, SVHC, Endocrine, Estrogen, Androgen, Structural analogy, QSAR, Priority setting, EINECS

## Abstract

**Background:**

The European chemicals’ legislation REACH aims to protect man and the environment from substances of very high concern (SVHC). Chemicals like endocrine disruptors (EDs) may be subject to authorization. Identification of (potential) EDs with regard to the environment is limited because specific experimental assessments are not standard requirements under REACH. Evidence is based on a combination of in vitro and in vivo experiments (if available), expert judgement, and structural analogy with known EDs.

**Objectives:**

The objectives of this study are to review and refine structural alerts for the indication of potential estrogenic and androgenic endocrine activities based on in vitro studies; to analyze in vivo mammalian long-term reproduction studies with regard to estrogen- and androgen-sensitive endpoints in order to identify potential indicators for endocrine activity with regard to the environment; to assess the consistency of potential estrogenic and androgenic endocrine activities based on in vitro assays and in vivo mammalian long-term reproduction studies and fish life-cycle tests; and to evaluate structural alerts, in vitro assays, and in vivo mammalian long-term reproduction studies for the indication of potential estrogenic and androgenic endocrine disruptors in fish.

**Results:**

Screening for potential endocrine activities in fish via estrogenic and androgenic modes of action based on structural alerts provides similar information as in vitro receptor-mediated assays. Additional evidence can be obtained from in vivo mammalian long-term reproduction studies. Conclusive confirmation is possible with fish life-cycle tests. Application of structural alerts to the more than 33,000 discrete organic compounds of the EINECS inventory indicated 3585 chemicals (approx. 11%) as potential candidates for estrogenic and androgenic effects that should be further investigated. Endocrine activities of the remaining substances cannot be excluded; however, because the structural alerts perform much better for substances with (very) high estrogenic and androgenic activities, there is reasonable probability that the most hazardous candidates have been identified.

**Conclusions:**

The combination of structural alerts, in vitro receptor-based assays, and in vivo mammalian studies may support the priority setting for further assessments of chemicals with potential environmental hazards due to estrogenic and androgenic activities.

**Electronic supplementary material:**

The online version of this article (doi:10.1186/s12302-016-0094-5) contains supplementary material, which is available to authorized users.

## Background

The European chemicals’ legislation REACH (EU 1907/2006) [1] aims to protect man and the environment from substances of very high concern (SVHC). Chemicals with (very) persistent, (very) bioaccumulative, and toxic properties (PBT and vPvB compounds), substances that are carcinogenic, mutagenic, and toxic to reproduction (CMR compounds), as well as chemicals of equivalent concern like endocrine disruptors (EDs), see Box 1, and sensitizers may be subject to authorization.

**Box 1:** Definition of endocrine disruptors

An **Endocrine Disruptor** is an *“exogenous substance or mixture that* ***alters function(s) of the endocrine system*** *and* ***consequently*** *causes* ***adverse health effects*** *in an intact organism, or its progeny, or (sub)populations,”* according to the widely accepted WHO/IPCs definition. The REACH regulation does not use the term “Endocrine Disruptors” but refers to “substances—such as those **having endocrine disrupting properties** […]—for which there **is scientific evidence of probable serious effects** to human health or the environment which **give rise to an equivalent level of concern**” compared to CMR and PBT/vPvB substances (Art 57f). However, the WHO/IPCS definition has been used for the identification of EDs as substances of very high concern and was confirmed to be the base for SVHC identification by the European Commission in its communication with regard to Endocrine Disruptors in June 2016 (http://ec.europa.eu/health/endocrine_disruptors/docs/com_2016_350_en.pdf)	

The Roadmap 2020, proposed by the European Commission in February 2013, asks all European Member States and the European Chemicals Agency (ECHA) to consider by 2020 which hazardous chemicals may be SVHC [2]. In this context, identification of potential EDs is restricted because specific experimental assessments are not standard requirements under REACH.

The OECD tiered conceptual framework for testing and assessment of EDs [3] involves OECD test guidelines and standardized test methods that can be used to evaluate chemicals for endocrine disruption. The guidance provides five levels of mammalian and non-mammalian toxicology using existing data and non-test information (level 1), in vitro (level 2) and in vivo (level 3) assays of selected endocrine mechanisms and pathways, in vivo assays providing data on adverse effects on endocrine relevant endpoints (level 4), and in vivo effects over more extensive parts of the life cycle of the organisms (level 5). Respective data are, however, available only for a limited number of chemicals.

Many currently known EDs operate via sexual endocrine pathways, and estrogenic (E) and androgenic (A) EDs (EA-EDs), agonists and antagonists, are thus an important group of possible SVHC candidates under REACH. The screening for potential EDs with regard to the environment to identify SVHC candidates is currently based on a combination of observations from in vitro and/or in vivo experiments (if available), structural analogy with known EDs, and expert judgement. The evaluation of information inherent to the chemical structures of the compounds is an appropriate starting point without the immediate need for animal experiments. Key to chemical structure-based approaches is the paradigm that similar chemical structures have similar properties and effects. However, chemical similarity is a complex and context-dependent phenomenon. Regarding estrogenic or androgenic activities, similar chemicals share one or several chemical patterns related to EA receptor interactions, the so-called structural alerts [4–19]. If a structural alert for potential ligands of EA receptors, acting as agonists or antagonists, is present in a compound, there is evidence that it may be EA-ED and closer inspection may be warranted.

The present study addresses screening for EA-EDs in fish based on evidence from structural alerts and in vitro and in vivo toxicological assays:

- review and refinement of structural alerts for the indication of potential estrogenic and androgenic endocrine activities based on in vitro studies;
- analysis of in vivo mammalian long-term reproduction studies with regard to estrogen- and androgen-sensitive endpoints in order to identify potential indicators for endocrine activity with regard to the environment;
- consistency of potential estrogenic and androgenic endocrine activities based on in vitro assays and in vivo mammalian long-term reproduction studies and fish life-cycle tests; and
- evaluation of structural alerts, in vitro assays, and in vivo mammalian long-term reproduction studies for the indication of potential estrogenic and androgenic endocrine disruptors in fish.

The results of this study have been used to develop a computerized screening tool for the German Environment Agency (Umweltbundesamt, UBA) to identify potential EA-EDs with regard to the environment based on structural alerts related to binding to estrogen and androgen receptors. Other endocrine mechanisms, for example, interference with thyroid hormones or steroidogenesis, damage of the corticosteroid system or the immune system, or epigenetic effects, are not regarded in this study.

Elements of the screening tool are used for a computerized mass screening performed by ECHA [20]. The mass screening is part of a common screening approach developed by ECHA and Member States aiming at identifying those substances which might be subject to further evaluation or risk management measures. The mass screening includes hazard-based indicators such as structural alerts but also considers potential emissions to man and the environment. In a second step, substances identified during mass screening are manually screened by member states, taking into account additional information, with the aim to decide whether or not further information is needed to conclude on the hazard potential. If information is missing to conclude on the endocrine-disrupting properties, the chemical may become subject to substance evaluation under REACH, a process permitting to request non-standard information on endocrine-disrupting properties. If substance evaluation confirms endocrine disruption, further regulatory action such as identification as SVHC and eventually restriction or the need for application for authorization might be triggered.

## Results and discussion

Screening for potential endocrine disruptors in fish was approached based on evidence from structural alerts and in vitro and in vivo toxicological assays. First, structural alerts for the indication of potential estrogenic and androgenic endocrine activities were reviewed and refined based on in vitro studies. Then, consistencies between in silico structural alerts, in vitro assays, and in vivo mammalian long-term reproduction studies and fish life-cycle tests were assessed. Finally, the model was applied to EINECS to search for potential candidates for estrogenic and androgenic effects with regard to the environment.

### Review and refinement of structural alerts for the indication of potential estrogenic and androgenic endocrine activities based on in vitro studies

#### In vitro data collection

Data from in vitro tests on estrogenic and androgenic effects (the so-called sexual endocrine effects) were retrieved from established databases [4, 21] and the literature (see Additional file 1). The focus was on tests based on receptor binding and receptor activation in cell cultures measured via reporter gene activation or cell proliferation:

- Competitive ligand-binding assays measure the binding affinity of a substance to an (isolated) receptor.
- Reporter gene assays measure activities of intact cells as a result of receptor-binding, for example mRNA, protein. The cells are transiently or permanently transfected with reporter gene systems.
- Cell proliferation assays measure cell proliferation triggered by receptor binding.

The collected dataset covers EA endocrine agonistic and antagonistic activities of 744 discrete chemicals. Substances were categorized based on their in vitro potencies in individual tests, relative receptor-binding affinities, or effect concentrations relative to positive controls, ethinylestradiol (estrogenic) or testosterone (androgenic), respectively (Table 1). If several test results were available for the same compound, we used the highest reported in vitro potency. For a detailed description of the experimental data used to identify receptor-mediated EA endocrine activities of substances, see the “Methods” section and Additional file 1.

**Table 1:** Overview of relative in vitro EA endocrine potencies (agonistic and antagonistic) of test substances

Activity	Relative potency	Number of substances
Very high	≥1	35
High	0.1–1	66
Moderate	0.001–0.1	110
Weak	0.00001–0.001	96
Very weak/inactive	<0.00001	437
Total number of substances		744

Relative potency: substances were categorized based on their in vitro potencies in individual tests, relative receptor-binding affinities, or effect concentrations relative to positive controls, ethinylestradiol (estrogenic) or testosterone (androgenic), respectively

Many chemicals are potential ligands of both, the estrogen and androgen receptors. Substantial similarity of EA receptors regarding their binding sites can be assumed. Only a few chemical classes interact only with one of these receptor families, such as phthalates [5]. Because any kind of binding of xenobiotics to estrogen or androgen receptors is undesirable, we pooled the results from studies with both receptor types for the identification of relevant structural alerts. With the same reasoning, we did not differentiate between agonistic and antagonistic activities, since binding to the receptor is a prerequisite for both activities.

#### Analysis of available structural alerts

The collected dataset of estrogenic and androgenic activities in vitro provided a comprehensive basis to analyze and define structural alerts that can be used to screen for potential EA-EDs (Table 2). First, we tested the applicability of structural alerts reported in the literature [1–17] like, for example, steroids, phytoestrogens, diphenylmethanes, biphenyls, bisphenols, phthalates, and alkylphenols (for details, see Additional file 2). The existing structural alerts identified many potential EA-EDs, but also resulted in a considerable fraction of false negatives with active EA-EDs not detected. False negatives are particularly critical because they could result in severe problems if adequate precaution was then not taken.

**Table 2:** Performance of structural alerts for EA-EDs based on the number of substances with endocrine activity that contain the structural alert [Actives (true positives)] relative to the number of substances without endocrine activity that contain the same structural alert [Inactives (false positives)] (Table extracted from the project report [26])

Structural alert	Structure	SMILES	Actives (true pos.)	Inactives (false pos.)	% actives	Relevance for EA-ED screening	Comments	Ref.
SA 01		C1CCC4C(C1)C2C(C3C(CC2)CCC3)CC4	56	8	87	X		[4, 7, 8]
SA 02		c1ccc3c(c1)C2C(CCCC2)CC3	19	0	100		Covered by SA 01	[11]
SA 03		c1ccc2c(c1)C(C(C)CC2)C	21	0	100	X		[40]
SA 04		c1cccc(c1)C=Cc2ccc(cc2)O	14	1	93	X		[4]
SA 05		c1cccc(c1)CCc2ccc(cc2)O	17	2	89	X		[4, 7]
SA 06		c1cccc(c1)CCc2ccccc2	18	8	69		More false positives than SA 05	[5, 8]
SA 07		c1cccc(c1)C(=Cc2ccccc2)c3ccccc3	8	0	100	X		[4]
SA 08		C3(c1ccccc1)Oc2c(cccc2)C(C=3)=O	8	7	53			[4]
SA 09		C3(c1ccccc1)Oc2c(cccc2)C(C3)=O	5	4	56			[4]
SA 10		C3(c1ccccc1)C(c2c(cccc2)OC3)=O	0	0				[4]
SA 11		C3(c1ccccc1)=Cc2c(cccc2)OC3=O	1	0	100	X		[4]
SA 12a		C(c1ccccc1)CC(c2ccccc2)=O	7	4	64			[4]
SA 12b		O=C(c(cccc1)c1)C=Cc(cccc2)c2	12	8	60	X		[4]
SA 13		C2C(c1c(cc(cc1O([H]))O([H]))CCCCCCCCCC2)=O	0	0				[4]
SA 14		c1cccc(c1)Cc2ccccc2	46	35	57			[4, 5]
SA 15a		c1cccc(c1O([H]))C(c2ccccc2)=O	3	7	30			[4, 5, 7, 16]
SA 15b		c1(cccc(c1)C(c2ccccc2)=O)O[H]	0	2	0			[4, 5, 7, 16]
SA 15c		c1c(ccc(c1)C(c2ccccc2)=O)O[H]	4	1	80	X		[4, 5, 7, 16]
SA 16		c1cccc(c1)C(c2ccccc2)=C(Cl)Cl	6	0	100	X		[4, 18]
SA 17		c1cccc(c1)C(c2ccccc2)C	19	15	56	X	Coverage of, e.g., DDT analogues	[7, 17]
SA 18		c1ccccc1c2ccccc2	11	14	44	X		[5–7, 12, 13, 16, 19]
SA 19a		c1ccccc1c2c(cccc2)Cl	6	2	75	X		[4]
SA 19b		c1ccccc1c2cc(ccc2)Cl	4	3	57	X	Analogy with SA 19a and 19c	[4]
SA 19c		c1ccccc1c2ccc(cc2)Cl	6	3	67	X		[4]
SA 20a		c1ccccc1c2c(cccc2)O[H]	0	1	0			[4]
SA 20b		c1ccccc1c2cc(ccc2)O[H]	0	1	0			[4]
SA 20c		c1ccccc1c2ccc(cc2)O[H]	6	0	100	X		[4]
SA 21		c1ccc2c(c1)Oc3c(O2)cccc3	1	0	100	X		[12]
SA 22		c1ccc2c(c1)oc3c2cccc3	0	0				[12]
SA 23		c1ccc(c(c1)C(OC)=O)C(=O)OC	7	6	54	X		[5, 7, 18]
SA 24		c1cc(ccc1C(OC)=O)O[H]	16	2	89	X		[4, 5]
SA 25a		c1c(c(ccc1)O([H]))OC	3	3	50			[4]
SA 25b		O(C)c1cc(ccc1)O[H]	15	17	47			[4]
SA 25c		O(C)c1ccc(cc1)O[H]	7	3	70	X		[4]
SA 26		c1cc(ccc1)O[H]	131	98	57		Specified phenols: see SA 04, SA 05, SA 15, SA 20, SA 25, SA 34	[5, 7–11, 14–16, 18, 40]
SA 27		C1(=O)CCCCC1	7	4	64			[40]
SA 28		c1cccc(c1)C(c2ccccc2)c3ccccc3	1	5	17			[7]
SA 29		c1c(ccc(c1)S(c2ccc(cc2)O([H]))(=O)=O)O[H]	0	1	0			[7]
SA 30a		c1c(ccc(c1)Oc2c(cc(cc2)Cl)Cl)Cl	2	0	100	X		[18]
SA 30b		c1c(ccc(c1)Oc2c(cc(cc2)Cl)Cl)N(=O)=O	4	0	100	X		[18]
SA 31a		c1cccc(c1)CN2C(c3c(C2=O)cccc3)=O	1	1	50			[7]
SA 31b		c1c(ccc(c1)CN2C(c3c(C2=O)cccc3)=O)N(=O)=O	0	0				[7]
SA 31c		c1c(ccc(c1)CN2C(c3c(C2=O)cccc3)=O)O[H]	1	0	100	X		[7]
SA 32a		c1cccc(c1)OP(=O)(OC)OC	1	0	100	X		[7]
SA 32b		c1cccc(c1)OP(=S)(OC)OC	3	1	75	X		[7]
SA 33		c1nc(N)nc(N)n1	0	6	0			[7]
SA 34a		[H]Oc1ccc(C([H])C)cc1	39	15	72	X	Specified from SA 26	
SA 34b		[H]Oc1ccc(C(C)(C)C([H]))cc1	7	9	44		Specified from SA 26	
SA 34c		[H]Oc1c([H])c([H])c(C([H])C)c([H])c([H])1	23	5	82	X	Specified from SA 26	
SA 34d		[H]Oc1c([H])c([H])c(C(C)(C)C([H]))c([H])c([H])1	6	2	75	X	Specified from SA 26	
SA 35		c(c(c(c(c1)ccc2)c2)ccc3)(c1)c3	13	8	62	X	Coverage of certain active polyaromatic compounds	
SA 36a		c1ccccc1CC=O	28	35	44			[7]
SA 36b		c1ccccc1CP=S	0	0				[18]
SA 37		c1ccccc1N2C(=O)CCC(=O)2	3	1	75	X		
SA 38		C1CCCCCCCCCCC1	3	2	60	X	Coverage of HBCDs	
SA 39		CCCCC([H])c1cc(OC)ccc1	5	1	83	X		
SA 40		CCCCC([H])c1ccc(OC)cc1	9	0	100	X		
SA 41		c1c(C(F)(F)F)cccc1NC=O	4	0	100	X		
SA 43		c1ccccc1COc2ccccc2	16	14	53	X		
SA 46		C12C=CC(C2)CC1	8	6	57	X	Coverage of norbornenes	
SA 47		C12CCC(C2)CC1	6	2	75	X	Coverage of norbornenes	
SA 48a		c1(Br)ccccc1Oc1ccccc1	14	2	88	X	Coverage of BDEs	
SA 48b		c1c(Br)cccc1Oc1ccccc1	11	3	79	X	Coverage of BDEs	
SA 48c		c1cc(Br)ccc1Oc1ccccc1	17	3	85	X	Coverage of BDEs	
SA 49		c12ccccc2CCC1	3	1	75	X		

Relevant structural alerts (X in column “Relevance for EA-ED screening”) are present more in substances with endocrine activity than in substances without endocrine activity

#### Development of improved structural alerts

To improve the detection of potential EA-EDs, we identified more structural alerts by systematic inspections of the chemical structures of substances with endocrine activity, for example, brominated diphenyl ethers (BDEs) and hexabromocyclododecanes (HBCDDs) that are antagonists in EA receptor-binding assays [22]. Furthermore, we looked for possible refinements of established structural alerts. The phenolic ring structure is an essential element of many EA-EDs; however, without further specifications it is not an indicative structural alert. Established “phenol” alerts explicitly address, for example, DES, biphenylols, or parabens [4, 5, 7]. For additional improvements, we suggest *p*-alkyl substitution pattern to increase the detection of actives from less than 60% to more than 70% (see Table 2, SA 34 as compared to SA 26).

The performance of existing and new structural alerts was evaluated with the collected dataset of estrogenic and androgenic activities in vitro based on the number of substances with endocrine activity that contain the structural alert (“true positives”) relative to the number of substances without endocrine activity that contain the same structural alert (“false positives”). Table 2 lists the structural alerts together with their occurrences in either active or inactive substances. Generally, a structural alert is considered a relevant EA-ED indicator (X in column “relevance for EA-ED screening”) if it could be found more often in active substances than in inactive substances. An empirical threshold of two-thirds was used to balance between too many “false positives” and too many “false negatives”. A few structural alerts with lower percentage of actives were included when either addressing very potent EA-EDs or covering large numbers of actives. Some structural alerts with high percentage of actives were not included because very similar structural alerts were available with even better performance (see Table 2, for example SA 6 as compared to SA 4 and 5). Some structural alerts could not be evaluated because they are not represented by the chemicals of the collected dataset of estrogenic and androgenic activities in vitro (see Table 2, for example SA 10, 13, 22, 31b, 36b).

The structural alerts (Table 2) classify 257 of 307 (84%) substances with endocrine activity in vitro as “true positives”. The presence of a relevant structural alert thus clearly indicates potential EA receptor agonists and antagonists. These are priority pollutants to undergo further assessments of their potential for endocrine activities. At the same time, the structural alerts indicate 100 of 437 (23%) substances without endocrine activity in vitro as false positives. False-positive predictions may lead to additional testing to show the absence of EA activities. Concern is related to false negatives [50 of 307 (16%)], chemicals with EA endocrine activities in vitro, but not recognized by structural alerts. Closer inspection of the 50 chemicals with false-negative predictions of EA-ED activities (for details, see Additional file 3) reveals 16 compounds with only (very) weak in vitro potencies according to the in vitro test results. With regard to prioritization of hazardous substances, these compounds may be considered less relevant. More significant are outliers with moderate (*n* = 26), high (*n* = 6), and very high (*n* = 2) in vitro potencies. Notably, among the eight (very) highly potent compounds are six antiandrogens like linuron and cypermethrin. For example, linuron is a weak in vivo AR antagonist that causes antiandrogenic activity via enzymatic pathway inhibition [23] and thyreotoxicity [24]. Impairment of thyroidal function has profound effects on fetal development and postnatal maturation and may mask EA-mediated effects. The 26 false-negative outliers of moderate in vitro potency are chemically diverse, including several pharmaceuticals and pesticides. The search for relevant structural alerts to detect these compounds was not successful. Another influential factor may be related to uncertainties of individual test results. Although the data were taken from peer-reviewed literature and quality-checked databases, “outliers” cannot be excluded.

It is important to note that the structural alerts are only indicators of the potential of chemical substances to be ligands of estrogen or androgen receptors. It is not possible to conclude mode and strength of potential agonistic or antagonistic activities. Furthermore, chemicals acting by other endocrine pathways will not be recognized.

### Analysis of in vivo mammalian long-term reproduction studies with regard to estrogen- and androgen-sensitive endpoints

**Fig. 1:**
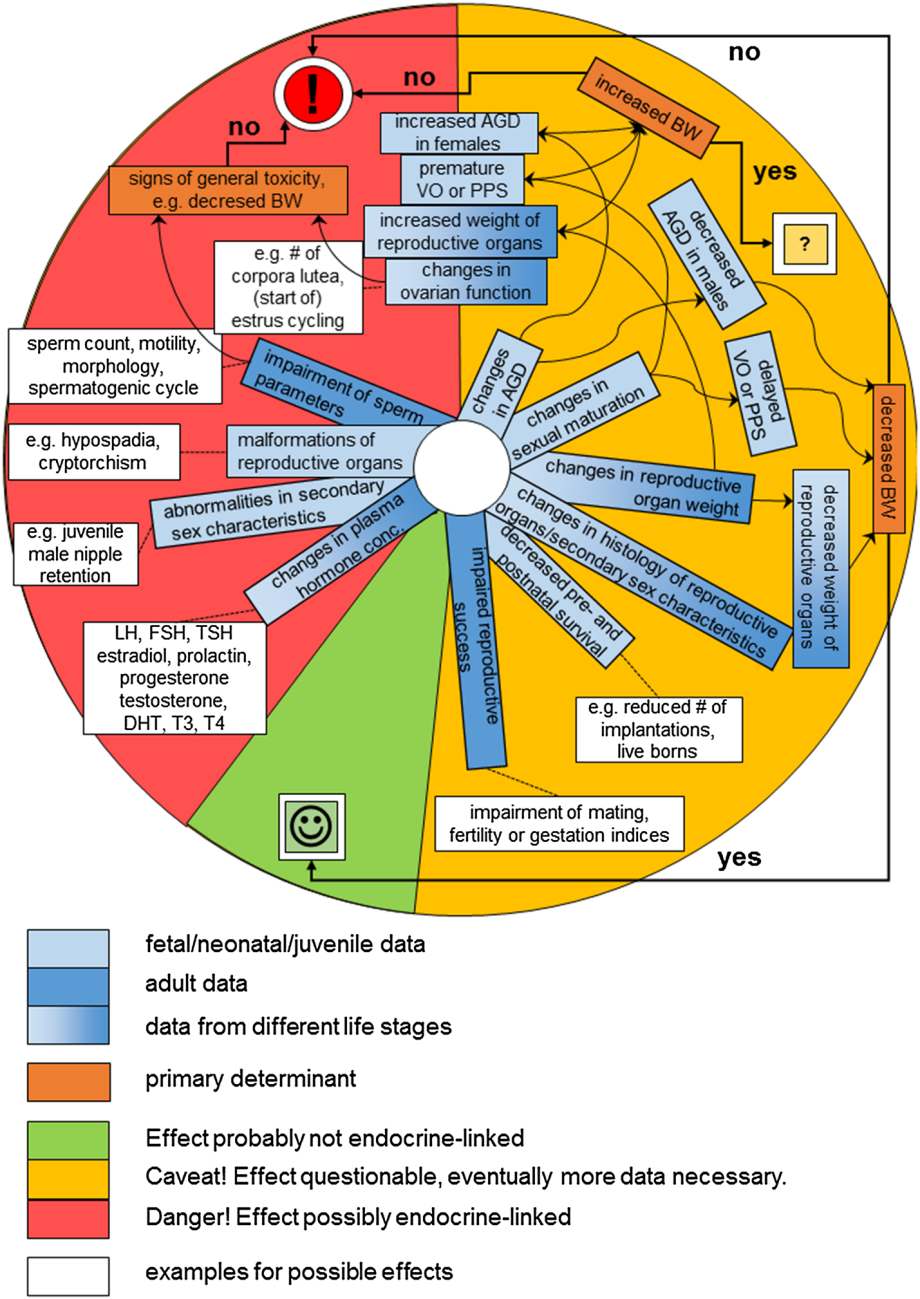
Scheme for differentiation between general reproductive toxicity and interactions with endocrine systems based on a weight-of-evidence approach. *AGD* anogenital distance, *BW* body weight, *DHT* dihydrotestosterone, *FSH* follicle-stimulating hormone, *LH* luteinizing hormone, *PPS* preputial separation, *T3* tri-iodothyronine, *T4* thyroxine, *TSH* thyroid-stimulating hormone, *VO* vaginal opening

Long-term in vivo studies of the effects on reproduction and development of mammals may include effects on endocrine systems. Symptoms like estrus cycle irregularities, reduced reproduction rate, or delayed sexual maturation may be due to systemic toxic effects, for example seen as decreased body weight, but also due to interferences with the endocrine system [25]. Although causal links between the observed effects and endocrine disorders may be difficult to establish (Fig. 1), evaluation of existing information from repeated dose and multi-generation in vivo studies indicated several parameters related to potential endocrine disruption [26] and can thus be used to inform about potential endocrine disruption with regard to the environment:

- malformation of reproductive organs, for example, agenesis/atrophic changes of testes, epididymis, and prostate;
- malformation and/or deviation of secondary sex characteristics, for example, hypospadia, male rodent nipple retention, and increased female anogenital distance;
- changes in serum hormone concentrations, for example, luteinizing hormone (LH), follicle-stimulating hormone (FSH), estrogen, and dihydrotestosterone (DHT); and
- effects on the onset of puberty/sexual maturation, for example, delayed preputial separation.

As depicted in Fig. 1, deviation of only one parameter is in most cases not sufficient to conclude an endocrine activity. First, it should be evaluated if a secondary effect can be excluded, for example, due to changes in body weight. Second, it should be checked if further parameters are affected and the conclusion should be drawn based on a weight-of-evidence approach.

Reproductive toxicity data and subacute to chronic repeated dose toxicity studies were retrieved from the FedTex (http://cefic-lri.org/lri_toolbox/fedtex/) and the RepDose (http://www.fraunhofer-repdose.de/) databases. The FedTex and RepDose data were selected from the available literature based on the level of detail regarding, for example, description of study design and details given for results. The data were double-checked by a second expert. Screening for chemicals with positive effect parameters related to potential endocrine disruption resulted in a list of 240 organic compounds. These compounds are reproductive toxicants with a certain likelihood of endocrine disruption. An indication of the EA-EDs among the 240 compounds was obtained from a comparison with results from receptor-based in vitro assays. We observed about half of them to interact with EA receptors, indicating true positives that are toxic for reproduction and likely to have endocrine activities in mammals. Such substances are also potential endocrine disruptors with regard to the environment and further assessment of effects in fish might be needed. The other half of the 240 compounds are inactive in receptor-based in vitro assays and probably not EA-EDs in mammals.

### Consistency of potential estrogenic and androgenic endocrine activities based on in vitro assays and in vivo mammalian long-term reproduction studies and fish life-cycle tests

The predictive power of in vitro receptor binding and in vivo toxicity in mammals for endocrine activity in fish was assessed by comparing their outcome with results of reproduction and biomarker studies with fish, focusing on sexual endocrine endpoints, from an UBA report [27] and the literature cited therein (see Table 3). Population-relevant endpoints were addressed by full life-cycle and two-generation tests (FLC) focusing on the effect parameters reproduction (fertilized eggs, fertility rate), sexual development, and generation time. Partial life-cycle tests also address population-relevant effects on sexual development and reproduction. Indicative biomarkers for endocrine effects were investigated in the short-term fish screening assay (FSA) including vitellogenin and steroid hormone levels in blood. Regarding fish in vivo data, in total 22 studies (fish full life-cycle, partial life-cycle, and fish screening assays) were available for 12 substances. The effects of these 12 substances on fish were compared to the effects on mammals and in in vitro tests (Table 3). Only for eight of them, also reproductive toxicity data for rodents were available. Receptor-based in vitro data could be retrieved for all the 12 substances. There is a remarkably good consistency of the data from fish and mammalian tests. The receptor-based in vitro activities support comparable sexual endocrine pathways of toxic action in fish and mammals. Combined assessment of data obtained in in vitro assays and in vivo fish tests reveals information on the underlying mechanism of action of the tested substance in fish, for example, reduced fertilization rate due to inhibition of androgen receptor activity in males.

**Table 3:** Comparison of in vivo long-term reproduction studies with mammals and fish with results from receptor-based in vitro assays

Compound	CAS no.	Mechanism	Mammalian reproductive toxicology	Fish in vivo effects			Reporter gene (cell) assays			Receptor-binding studies	
				Fish full and partial life-cycle (FLC) test		Fish screening assay (FSA)	Potency relative to positive control			Potency relative to positive control	
			In vivo ED effect LOAEL	NOEC/EC_10_ population	NOEC indicator parameter	Effect	ER agonist	AR agonist	AR antagonist	ER	AR
17-α Ethinylestradiol	57-63-6	ER agonist	Pos.-control in screening assays (OECD 440)	0.3 ng/L, fertilization rate, delayed oviposition [41]	1 ng/L, ovotestis [42]	VTG increase [27]	Very active [43]		No [28]	Very active [5]	Moderate [21]
17-β Estradiol	50-28-2	ER agonist	0.0025 mg/kg BW	2.9 ng/L, fertilization rate, juvenile growth [44]	2.9 ng/L, VTG induction, males [44]		Very active [45]	Moderate [28]	Active [18]	Very active [5]	Active [21]
Tamoxifen	10540-29-1	ER antagonist	0.00012 mg/kg BW	1.6 µg/L, fertilization rate [27]	1.6 µg/L, VTG decrease, females [27]	VTG decrease [27]	Moderate [43]		Moderate [46]	Very active [21]	Active [46]
Genistein	446-72-0	ER agonist, enzyme inhibitor: aromatase	1.25 mg/kg BW	1.3 µg/L, fertilization rate, delayed oviposition [27]			Moderate [47]			Very active [21]	Moderate [21]
Methyl-testosterone	58-18-4	AR agonist		10 ng/L, sex ratio [48]	<0.4 ng/L, VTG decrease, females [48]		Active [49]	Active [49]			Very active [21]
17β-Trenbolone	10161-33-8	AR agonist	1.68 mg/kg BW	3 ng/L, sex ratio [50]	<1 ng/L, histology: egg debris [50]	VTG and 11-keto-testosterone: no effect [27]		Active[28]			Very active [21]
Flutamide	13311-84-7	AR antagonist	2.5 mg/kg BW	189 µg/L, egg production [27]	435 µg/L, VTG decrease, females, 11-keto-testosterone: increase [27]	VTG no effect, 11-keto-testosterone: increase [27]		Weak [28]	Very active [28]		Moderate [21]
Bisphenol A	80-05-7	ER agonist	0.0002 mg/kg BW	390 µg/L, fertilization rate, delayed oviposition [51] 160 µg/L, hatching rate of F1 generation [52]	1 µg/L, histology: testis, VTG increase, males [52]	VTG increase, 11-keto-testosterone: decrease [27]	Moderate [53]	Very weak [54]	Active [18]	Moderate [5, 21]	Moderate [21]
4-*tert*-Pentylphenol	80-46-6	ER agonist		100 µg/L, fertilization rate, sex ratio [55]	<51 µg/L, VTG increase, males [55]				Active [18]	Moderate [5, 21]	Moderate [21]
4-*tert*-Octylphenol	140-66-9	ER agonist		11 µg/L, fertilization rate, delayed oviposition [27]		VTG increase, 11-keto-testosterone: decrease [27]	Moderate [56]	Very weak [57]	Active [18]	Moderate [5, 21]	Moderate [21]
4-Nonylphenol	84852-15-3	ER agonist	10 mg/kg BW	8.2 µg/L, sex ratio, survival rate [58]	4.2 µg/L, gonadosomatic index [58]		Moderate [59]	Very weak [60]	Very weak [60]	Moderate [21]	Moderate [21]
Prochloraz (DMI fungicide)	67747-09-5	Aromatase inhibitor		64 µg/L, sex ratio [61]	<16 µg/L, VTG increase, males [61]	VTG decrease [27]	Very weak [62]		Very weak [62]		

Mammalian reproductive toxicology from: http://cefic-lri.org/lri_toolbox/fedtex/, http://www.fraunhofer-repdose.de/

Relative potencies in receptor-based in vitro assays: substances were categorized based on their in vitro potencies in individual tests, relative receptor-binding affinities, or effect concentrations relative to positive controls, ethinylestradiol (estrogenic) or testosterone (androgenic), respectively (Table 1)

*LOAEL* lowest observed adverse effect level, *NOEC* no observed effect concentration, *ER* estrogen receptor, *AR* androgen receptor, *VTG* vitellogenin, *DMI fungicide* demethylation inhibitor (sterol synthesis inhibitor)

The substances identified as “endocrine active” in fish tests are reproductive toxicants in mammals and EA receptor ligands in in vitro assays (Table 3). There are no false-negative results; however, the relative potencies vary. For example, flutamide is a moderate AR agonist in in vivo fish FLC tests [27], very active in in vitro reporter gene assays [28], and causes malformations in epididymis, seminal vesicles, prostate, Cowper’s glands, and penis in rodents [29]. It furthermore affects male secondary sexual characteristics in terms of feminization, impairs spermatogenesis, and alters pituitary hormone concentrations [30].

### Evaluation of structural alerts, in vitro assays, and in vivo mammalian long-term reproduction studies for the indication of potential estrogenic and androgenic endocrine disruptors in fish

Comparative analyses of the agreement of chemical and toxicological approaches to the identification of potential EA-EDs in fish were based on receptor-based in vitro assays and in vivo mammalian long-term reproduction studies. The combined dataset includes 933 chemicals, with 693 having only in vitro data and 189 having only in vivo mammalian long-term reproduction data. For 51 substances, in vivo as well as in vitro data were available. The minor overlap of the in vivo and in vitro datasets with only 51 chemicals shows that for many in vivo reproductive toxicants in rodents, the corresponding in vitro results that could specify the relevant modes of action have not been published.

The results of pairwise comparisons of the in vivo mammalian long-term reproductive toxicities and in vitro classifications with structural alerts of potential EA-EDs were quantified in terms of accuracy (proportion of substances correctly classified), sensitivity (proportion of true positives correctly classified), and specificity (proportion of true negatives correctly classified); for details, see “Methods” section.

The structural alerts agree quite well (80% accuracy) with the in vitro classifications (Table 4). This was to be expected since the structural alerts were derived from in vitro data. 16.3% (50 of 307) of the active chemicals in vitro are false negatives not recognized by the structural alerts. From the inactive chemicals in vitro, 22.9% (100 of 437) have the structural alerts and thus are false positives. Notably, performance of the structural alerts is better for chemicals with higher activity in vitro, and (very) high activities are classified more accurately than moderate to (very) weak activities.

**Table 4:** Comparison of in vitro activities and structural alerts of potential EA-EDs (numbers of chemicals, *n* = 744)

	In vitro very high activity	In vitro high activity	In vitro moderate activity	In vitro weak activity	In vitro very weak or no activity	Σ
Structural alert = yes	33	60	84	80	100	357
Structural alert = no	2	6	26	16	337	387
Σ	35	66	110	96	437	744

Accuracy = 79.8% (overall agreement)

Sensitivity = 83.7% (→16.3% false-negative classifications)

Specificity = 77.1% (→22.9% false-positive classifications)

Comparison of the classifications of the 51 chemicals tested in vivo and in vitro (Table 5) shows that in vitro classifications always coincide with in vivo mammalian long-term reproductive toxicities. If we see in vitro activity, we always also see reproductive toxicity in vivo. Thus, based on these results, positive results in receptor-based in vitro assays are good indicators of possible reproductive toxicity in vivo and suggest that EA receptor binding may be involved in the reproductive toxicity of these compounds (24 of 48, corresponding to 50% true positives). At the same time, inactivity in vitro is not a good indicator for the absence of reproductive toxicity in vivo. The reproductive toxicity of compounds without EA receptor-binding potential is likely due to other modes of action.

**Table 5:** Comparison of in vivo mammalian long-term reproductive toxicities and in vitro activities of potential EA-EDs (numbers of chemicals, *n* = 51)

	In vitro very high activity	In vitro high activity	In vitro moderate activity	In vitro weak activity	In vitro very weak or no activity	Σ
In vivo active	6	6	11	1	24	48
In vivo inactive	0	0	0	0	3	3
Σ	6	6	11	1	27	51

Accuracy = 52.9% (overall agreement)

Sensitivity = 100% (→no false-negative classifications)

Specificity = 50.0% (→50% false-positive classifications)

Mammalian long-term reproductive toxicants: in vivo active: causing specific endpoints indicative for endocrine activity, in vivo inactive: not causing specific outcomes in the available study/studies

For an overview of the relationships between chemical and toxicological approaches to the identification of potential EA-EDs, we calculated classification statistics of pairwise comparisons of in vivo mammalian long-term reproductive toxicities, receptor-based in vitro activities, and structural alerts of potential EA-EDs. Table 6 summarizes the overall agreement, sensitivities, and specificities either for the full datasets (*n* = 744 or *n* = 240 for the receptor-based in vitro activities or the in vivo mammalian long-term reproductive toxicities, respectively) or the subset of compounds with in vitro and in vivo data (*n* = 51). We observe similar results for the relationships between in vitro activities and structural alerts, regardless of the size and composition of the datasets. The similar information content of the in vitro activities and the structural alerts is furthermore the reason for the almost identical overall agreement, sensitivities, and specificities of activities in vivo with either structural alerts or in vitro activities, with regard to the same subset of compounds (*n* = 51). Again, we observe no false-negative results, but >50% false positives. The agreement is much less for the relationships between the entire dataset of activities in vivo (*n* = 240) and structural alerts. This is due to the in vivo mammalian dataset representing multiple pathways of reproductive toxicity, while the structural alerts are limited to EA receptor interactions.

**Table 6:** Overall agreement, sensitivities, and specificities of in vivo mammalian long-term reproductive toxicities, in vitro activities, and structural alerts of potential EA-EDs

	*n*	Overall agreement (%)	Sensitivity (%)	Specificity (%)
In vitro/structural alerts (all compounds with in vitro data)	744	79.8	83.7	77.1
In vitro/structural alerts (all compounds with in vitro and in vivo data)	51	84.3	79.2	88.9
In vivo/in vitro (all compounds with in vitro and in vivo data)	51	52.9	50.0	100
In vivo/structural alerts (all compounds with in vitro and in vivo data)	51	49.0	45.8	100
In vivo/structural alerts (all compounds with in vivo data)	240	20.0	15.4	100

Dataset *n* = 744: all compounds with in vitro data, dataset *n* = 240: all compounds with in vivo data, dataset *n* = 51: all compounds with in vitro and in vivo data

The screening for potential EA-EDs based on structural alerts relative to in vitro classifications and in vivo mammalian long-term reproductive toxicities is shown in Table 7. Regarding first the chemicals with mammalian long-term reproductive toxicity in vivo and receptor-based in vitro assays, we see positive results from the three approaches confirming each other for 19 chemicals (marked in red). Five substances with in vivo mammalian long-term reproductive toxicity are identified in vitro but not from their chemical structures (marked in orange). Structural alerts classify another three compounds with activity in vivo but not in vitro (marked in orange). For the remaining 21 mammalian long-term reproductive toxicants, neither in vitro assays nor structural alerts provide evidence of interferences with EA endocrine receptors and, thus, other toxic pathways are more likely causing the reproductive toxicity.

**Table 7:**
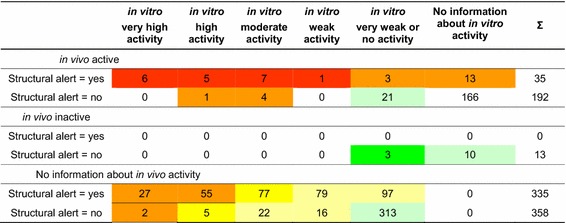
Screening for potential EA-EDs based on structural alerts relative to in vitro classifications and in vivo mammalian long-term reproductive toxicities (numbers of chemicals, *n* = 933)

Color code: red: very high probability of EA-EDs, orange: high probability of EA-EDs, yellow: moderate probability of EA-EDs, pale yellow: low probability of EA-EDs, pale green: very low probability of EA-EDs, green: unlikely EA-EDs

For 13 chemicals that are inactive in vivo, we observe no structural alerts and also the in vitro data suggest the absence of EA endocrine effects (marked in green).

Among the 744 chemicals in the combined dataset with in vitro data are 693 without information about in vivo mammalian long-term reproductive toxicity, 335 of them with structural alerts and 358 without structural alerts. The evaluation of their in vitro activities in combination with structural alerts indicates 84 chemicals (marked in orange) with high probability of EA-EDs in fish and further assessments are recommended. Lower probability of EA-EDs may be associated with the chemicals with moderate to minor activity in vitro and/or the absence of structural alerts [marked in (pale) yellow]. The priority for further assessments is very low for the 313 substances with only very weak or no activity in vitro and without structural alerts (marked in pale green).

### Application of structural alerts for the identification of potential EA-EDs in EINECS

The structural alerts for the identification of potential EA-EDs (Table 2) have been applied to EINECS (European inventory of existing commercial chemical substances) to test the performance on large numbers of diverse substances and to prioritize potential candidates for endocrine activity due to interactions with estrogen and androgen receptors. EINECS includes approx. 100,000 substances that were deemed to be on the European Community market between January 1, 1971 and September 18, 1981 [31]. Substances listed in EINECS are “existing chemicals” and considered phase-in substances under REACH.

For application to EINECS, the structural alerts (Table 2) have been implemented in a computerized screening tool (EDC-Scan).^1^ To support prediction confidence of the structural alerts in EDC-Scan, their applicability domain (AD) was defined based on atom-centered fragments (ACFs) [32]. The collected dataset of estrogenic and androgenic activities in vitro (see Additional file 1) was used as the training set for the determination of the AD of the structural alerts. If a chemical is within the AD because it is similar to the substances used to set up the model, predictions are considered most trustworthy. If a compound is outside the AD, reliability of estimates is low because it may act in different ways.

More than 33,000 discrete organic EINECS compounds are within the ACF-defined AD of the structural alerts for potential EA-EDs. Among them, structural alerts indicate 3585 chemicals (ca. 11%) as candidates with potential endocrine activity. Their chemical structures are principally able to interact with estrogen and androgen receptors as either agonists or antagonists. Due to the possibility that these chemicals may interact with EA receptors, they should be subject to further investigations regarding their potential for endocrine effects in fish.

Structural alerts have been detected in another 413 organic chemicals that are outside the ACF-defined AD of the structural alerts for the identification of potential EA-EDs. According to good modeling practice, these predictions are not valid because they are not supported by the activities of the compounds of the training set. Still, these 413 chemicals contain structural elements that may be able to interact with estrogen and androgen receptors. If no additional evidence for endocrine activity is available, such alerts would probably not be sufficient to trigger further regulatory action such as substance evaluation, although toxicological reasoning suggests that they are candidates for EA-ED activities.

Estrogenic and androgenic ED activities of the remaining substances cannot be excluded, but are less likely. Because the structural alerts perform much better for substances with (very) high estrogenic and androgenic activities (<10% false-negative classifications for strong binders) as compared to >15% false-negative classifications for (very) weak binders, there is reasonable probability that the candidates with potential endocrine activities based on structural alerts include the most hazardous EA-EDs.

## Conclusions

In vivo, in vitro, and in silico methods for the identification of potential EA-EDs with regard to the environment cover different adverse effects at the biomolecular and organism levels and indicate population-relevant effects. In vivo studies often summarize effects caused by multiple pathways of endocrine and other reproductive toxicities. In vitro assays may address specific modes of endocrine activities. Structural alerts represent the in vitro assays from which they were derived. The combination of in vivo and in vitro information with structural alerts provides complementary information for evidence-based assessments of potential EDs. Information obtained at each level may support the priority setting for further assessments of candidate chemicals with potential environmental hazards. Evidence for endocrine activity increases if structural alerts, in vitro activities, as well as in vivo information provide positive results.

Application of structural alerts for the identification of potential EDs to the EINECS inventory indicated 3585 chemicals (ca. 11%) as potential candidates for endocrine effects. Due to the possibility that these chemicals may interact with estrogen and androgen receptors, they should be subject to further investigations regarding their potential for endocrine activity with regard to the environment. Such substances are indicated during the mass screening performed by ECHA and manually screened by member states. They may become subject to regulatory actions if the screening evidence is substantiated by additional information.

## Methods

### Experimental data from in vitro assays

Data were collected from publicly available databases and the open literature for in vitro tests on estrogenic and androgenic receptor binding and receptor activation in cell cultures. Activities were measured via reporter gene activation or cell proliferation, for example, competitive ligand-binding assays, reporter gene assays with mammalian cells with endogenous estrogen receptors transiently or permanently transfected with luciferase reporter gene systems, for example, CALUX [33, 34] and MVLN, MCF-7, or HeLa cells [33, 35–38], yeast-based receptor gene tests stably transfected with a β-galactosidase reporter gene [14], and MCF-7 cell proliferation assay (E-Screen) [39].

1. US Food and Drug Administration Endocrine Disruptor Knowledge Base (FDA-EDKB): http://www.fda.gov/ScienceResearch/BioinformaticsTools/EndocrineDisruptorKnowledgebase/default.htm.
  The FDA-EDKB database contains data regarding binding to androgen as well as estrogen receptors. The ligand-binding studies were conducted and validated at NCTR (National Center for Toxicological Research, Jefferson, USA). The test results are reported as percent relative binding affinity (RBA) of the test substance relative to the respective positive standards (17R-methyl-^3^H]methyltrienolone (R1881) for AR and [^3^H]-17β-estradiol for ER).
  Androgen receptor (recombinant receptor): log (RBA) is given for 146 of 202 substances, ranging between 2.3 and −3.6. The remaining 56 substances are classified as non-binding.
  Estrogen receptor (isolated from rat uteri): log (RBA) is given for 131 of 232 substances, ranging between 2.6 and −4.5. The remaining 101 substances are classified as non-binding.
2. National Center for Toxicological Research Estrogen Receptor Binding Database (DSSTOX-NCTRER): http://www.epa.gov/ncct/dsstox/sdf_nctrer.html.
  The DSSTOX-NCTRER database contains relative receptor-binding affinities for 232 chemical substances based on ligand-binding studies with estrogen receptors (species: rat). Activities are classified in five “activity categories”. This dataset is identical with the estrogen receptor dataset of FDA-EDKB described above.
3. Data retrieved from primary literature:
  Primary literature was evaluated for test results from diverse in vitro assays (Table 8); 1155 test results were collected for 744 substances (data tables with references are provided in Additional file 1).

**Table 8:** Numbers of in vitro data retrieved from primary literature

In vitro assays	Number of test results
Reporter gene assays	
Androgen receptor activation, agonist	93
Androgen receptor activation, antagonist	395
Estrogen receptor activation, agonist ER alpha	202
Estrogen receptor activation, antagonist ER alpha	86
Estrogen receptor activation, agonist ER beta	62
Estrogen receptor activation, antagonist ER beta	7
Ligand-binding assays	
Receptor-binding AR	36
Receptor-binding ER alpha	246
Receptor-binding ER beta	28
Total	1155

*ER* estrogen receptor, *AR* androgen receptor

### Classification statistics

The results of pairwise comparisons of the in vivo, in vitro, and in silico classifications of potential EA-EDs were quantified in terms of accuracy (proportion of substances correctly classified), sensitivity (proportion of true positives correctly classified), and specificity (proportion of true negatives correctly classified):

$${\text{Accuracy}} = \frac{{\left( {{\text{TP}} + {\text{TN}}} \right)}}{\text{Tot}} \times 100$$

$${\text{Sensitivity}} = \frac{\text{TP}}{{{\text{TP}} + {\text{FN}}}} \times 100$$

$${\text{Specificity}} = \frac{\text{TN}}{{{\text{TN}} + {\text{FP}}}} \times 100$$

where TP: true positive, TN: true negative, FP: false positive, FN: false negative, and Tot: total number of compounds.

## Additional files

**Additional file 1.** Estrogenic and androgenic endocrine activities in vitro.

**Additional file 2.** Structural alerts.

**Additional file 3.** False negatives (not recognized by structural alerts).

## Footnotes

1 Within a follow-up project (UBA, FKZ 3714634120), the structural alerts for EA-EDs as well as other endocrine effects are being included in ChemProp (http://www.ufz.de/ecochem/chemprop).

## References

[1] European Commission (2006) Regulation (EC) no 1907/2006 of the European parliament and of the council of 18 December 2006 concerning the registration, evaluation, authorisation and restriction of chemicals (REACH), establishing a European chemicals agency, amending directive 1999/45/EC and repealing council regulation (EEC) no 793/93 and commission regulation (EC) no 1488/94 as well as council directive 76/769/EEC and commission directives 91/155/EEC, 93/67/EEC, 93/105/EC and 2000/21/EC. Brussels, Belgium

[2] European Commission (2013) Roadmap for SVHCs identification and implementation of REACH risk management measures from now to 2020. http://register.consilium.europa.eu/doc/srv?l=EN&f=ST%205867%202013%20INIT. Accessed 3 Nov 2015

[3] OECD (2012). Guidance document on standardised test guidelines for evaluating chemicals for endocrine disruption.

[4] US EPA. Distributed structure-searchable toxicity (DSSTox) database. https://www.epa.gov/chemical-research/distributed-structure-searchable-toxicity-dsstox-database. Accessed 21 Apr 2009

[5] Blair RM, Fang H, Branham WS, Hass BS, Dial SL, Moland CL, Tong W, Shi L, Perkins R, Sheehan DM (2000). The estrogen receptor relative binding affinities of 188 natural and xenochemicals: structural diversity of ligands. Toxicol Sci.

[6] Bradbury SP, Mekenyan OG, Ankley GT (1996). Quantitative structure–activity relationships for polychlorinated hydroxybiphenyl estrogen receptor binding affinity: an assessment of conformer flexibility. Environ Toxicol Chem.

[7] Fang H, Tong W, Branham WS, Moland CL, Dial SL, Hong H, Xie Q, Perkins R, Owens W, Sheehan DM (2003). Study of 220 natural, synthetic, and environmental chemicals for binding to the androgen receptor. Chem Res Toxicol.

[8] Hong H, Tong W, Fang H, Shi L, Xie Q, Wu J, Perkins R, Walker JD, Branham W, Sheehan DM (2002). Prediction of estrogen receptor binding for 58,000 chemicals using an integrated system of a tree-based model with structural alerts. Environ Health Perspect.

[9] Jordan VC, Mittal S, Gosden B, Koch R, Lieberman ME (1985). Structure–activity relationships of estrogens. Environ Health Perspect.

[10] Katzenellenbogen JA (1995). The structural pervasiveness of estrogenic activity. Environ Health Perspect.

[11] Klopman G, Chakravarti SK (2003). Structure–activity relationship study of a diverse set of estrogen receptor ligands (I) using MultiCASE expert system. Chemosphere.

[12] Krishnan V, Safe S (1993). Polychlorinated biphenyls (PCBs), dibenzo-*p*-dioxins (PCDDs), and dibenzofurans (PCDFs) as antiestrogens in MCF-7 human breast cancer cells: quantitative structure–activity relationships. Toxicol Appl Pharmacol.

[13] Navas JM, Alonso M, Casado S, Miranda C, Tarazona JV, Herradón B (2007) Structural features of ligands of the aryl hydrocarbon receptor: the case of the polybrominated biphenyl 209 (PBB-209). In: Poster presentation at SETAC Europe 17th annual meeting, Porto, Portugal

[14] Routledge EJ, Sumpter JP (1997). Structural features of alkylphenolic chemicals associated with estrogenic activity. J Biol Chem.

[15] Schmieder P, Aptula AO, Routledge EJ, Sumpter JP, Mekenyan OG (2000). Estrogenicity of alkylphenolic compounds: a 3-D structure–activity evaluation of gene activation. Environ Toxicol Chem.

[16] Schultz TW, Sinks GD, Cronin MTD (2002). Structure–activity relationships for gene activation oestrogenicity: evaluation of a diverse data set of aromatic chemicals. Environ Toxicol.

[17] Shi L, Tong W, Fang H, Tong W, Wu J, Perkins R, Blair RM, Branham WS, Dial SL, Moland CL, Sheehan DM (2001). QSAR models using a large diverse set of estrogens. J Chem Inf Comput Sci.

[18] Tamura H, Ishimoto Y, Fujikawa T, Aoyama H, Yoshikawa H, Akamatsu M (2006). Structural basis for androgen receptor agonists and antagonists: interaction of SPEED 98-listed chemicals and related compounds with the androgen receptor based on an in vitro reporter gene assay and 3 D-QSAR. Bioorg Med Chem.

[19] Waller CL, Minor DL, McKinney JD (1995). Using three-dimensional quantitative structure–activity relationships to examine estrogen receptor binding affinities of polychlorinated hydroxybiphenyls. Environ Health Perspect.

[20] ECHA (2015) A common screening approach for REACH and CLP processes. http://echa.europa.eu/documents/10162/19126370/common_screening_approach_en.pdf. Accessed 15 July 2016

[21] FDA. Endocrine disruptor knowledge base. http://www.fda.gov/ScienceResearch/BioinformaticsTools/EndocrineDisruptorKnowledgebase/default.htm. Accessed 21 Apr 2009

[22] Hamers T, Kamstra JH, Sonneveld E, Murk AJ, Kester MHA, Andersson PL, Legler J, Brouwer A (2006). In vitro profiling of the endocrine-disrupting potency of brominated flame retardants. Toxicol Sci.

[23] Webster TMU, Perry MH, Santos EM (2015). The herbicide linuron inhibits cholesterol biosynthesis and induces cellular stress responses in brown trout. Environ Sci Technol.

[24] Schmutzler C, Ambrugger P, Huhne K, Grüters A, Köhrle J (2004). Endocrine disrupters inhibit human thyroid peroxidase activity. Exp Clin Endocrinol Diabetes.

[25] Lewin G, Escher SE, van der Burg B, Simetska N, Mangelsdorf I (2015). Structural features of endocrine active chemicals—a comparison of in vivo and in vitro data. Reprod Toxicol.

[26] Müller M, Wenzel A, Nendza M, Lewin G (2009) Assessment and regulation of environmental hormones (sub-project 5): development of structure- and risk-based methods for the identification of chemicals with suspected endocrine disrupting activities for the prioritisation of chemicals as part of the authorisation procedure under REACH. Report: Forschungsprojekt im Auftrag des Umweltbundesamtes, FuE-Vorhaben FKZ 206 67 448/05. Dessau-Roßlau, Germany

[27] Teigeler M, Knacker T, Schäfers C (2007) Charakterisierung endokrin vermittelter Wirkungen in Fischen: Relevante Parameter für die Entwicklung einer neuen OECD-Testmethode und die Anwendung in der gesetzlichen Umweltrisikobewertung. Report: Forschungsprojekt im Auftrag des Umweltbundesamtes, FuE-Vorhaben FKZ 206 67 470. Dessau-Roßlau, Germany

[28] Bovee TFH, Helsdingen RJR, Hamers ARM, van Duursen MBM, Nielen MWF, Hoogenboom RLAP (2007). A new highly specific and robust yeast androgen bioassay for the detection of agonists and antagonists. Anal Bioanal Chem.

[29] McIntyre BS, Barlow NJ, Foster PM (2001). Androgen-mediated development in male rat offspring exposed to flutamide in utero: permanence and correlation of early postnatal changes in anogenital distance and nipple retention with malformations in androgen-dependent tissues. Toxicol Sci.

[30] Miyata K, Yabushita S, Sukata T, Sano M, Yoshino H, Nakanishi T, Okuno Y, Matsuo M (2002). Effects of perinatal exposure to flutamide on sex hormones and androgen-dependent organs in F1 male rats. J Toxicol Sci.

[31] European Commission (1990) EINECS (European inventory of existing commercial chemical substances). O.J. C 146A, (15.6.1990)

[32] Kühne R, Ebert RU, Schüürmann G (2009). Chemical domain of QSAR models from atom-centered fragments. J Chem Inf Model.

[33] Bonefeld-Jorgensen EC, Long M, Hofmeister MV, Vinggaard AM (2007). Endocrine-disrupting potential of bisphenol A, bisphenol A dimethacrylate, 4-*n*-nonylphenol, and 4-*n*-octylphenol in vitro: new data and a brief review. Environ Health Perspect.

[34] Legler J, van den Brink CE, Brouwer A, Murk AJ, van der Saag PT, Vethaak AD, van der Burg B (1999). Development of a stably transfected estrogen receptor-mediated luciferase reporter gene assay in the human t47d breast cancer cell line. Toxicol Sci.

[35] Bonefeld-Jorgensen EC, Grünfeld HT, Gjermandsen IM (2005). Effect of pesticides on estrogen receptor transactivation in vitro: a comparison of stable transfected MVLN and transient transfected MCF-7 cells. Mol Cell Endocrinol.

[36] Gutendorf B, Westendorf J (2001). Comparison of an array of in vitro assays for the assessment of the estrogenic potential of natural and synthetic estrogens, phytoestrogens and xenoestrogens. Toxicology.

[37] Jausons-Loffreda N, Balaguer P, Roux S, Fuentes M, Pons M, Nicolas JC, Gelmini S, Pazzagli M (1994). Chimeric receptors as a tool for luminescent measurement of biological activities of steroid hormones. J Biolumin Chemilumin.

[38] Pons M, Gagne D, Nicolas JC, Mehtali M (1990). A new cellular model of response to estrogens: a bioluminescent test to characterize (anti) estrogen molecules. Biotechniques.

[39] Soto AM, Sonnenschein C, Chung KL, Fernandez MF, Olea N, Serrano FO (1995). The e-screen assay as a tool to identify estrogens: an update on estrogenic environmental pollutants. Environ Health Perspect.

[40] Klopman G, Chakravarti SK (2003). Screening of high production volume chemicals for estrogen receptor binding activity (II) by the MultiCASE expert system. Chemosphere.

[41] Schäfers C, Teigeler M, Wenzel A, Maack G, Fenske M, Segner H (2007). Concentration- and time-dependent effects of the synthetic estrogen, 17α-ethinylestradiol, on reproductive capabilities of the zebrafish, Danio rerio. J Toxicol Environ Health A.

[42] Länge R, Hutchinson TH, Croudace CP, Siegmund F, Schweinfurth H, Hampe P, Panter GH, Sumpter JP (2001). Effects of the synthetic estrogen 17α-ethinylestradiol on the life-cycle of the fathead minnow (*Pimephales promelas*). Environ Toxicol Chem.

[43] Jungbauer A, Beck V (2002). Yeast reporter system for rapid determination of estrogenic activity. J Chromatogr B Analyt Technol Biomed Life Sci.

[44] Seki M, Yokota H, Maeda M, Kobayashi K (2005). Fish full life-cycle testing for 17β-estradiol on medaka (*Oryzias latipes*). Environ Toxicol Chem.

[45] Schreurs RHMM, Sonneveld E, Jansen JHJ, Seinen W, van der Burg B (2005). Interaction of polycyclic musks and UV filters with the estrogen receptor (ER), androgen receptor (AR), and progesterone receptor (PR) in reporter gene bioassays. Toxicol Sci.

[46] Yamasaki K, Sawaki M, Noda S, Muroi T, Takakura S, Mitoma H, Sakamoto S, Nakai M, Yakabe Y (2004). Comparison of the Hershberger assay and androgen receptor binding assay of twelve chemicals. Toxicology.

[47] Hasenbrink G, Sievernich A, Wildt L, Ludwig J, Lichtenberg-Frate H (2006). Estrogenic effects of natural and synthetic compounds including tibolone assessed in *Saccharomyces cerevisiae* expressing the human estrogen alpha and beta receptors. FASEB J.

[48] Seki M, Yokota H, Matsubara H, Maeda M, Tadokoro H, Kobayashi K (2004). Fish full life-cycle testing for androgen methyltestosterone on medaka (*Oryzias latipes*). Environ Toxicol Chem.

[49] Ma R, Cotton B, Lichtensteiger W, Schlumpf M (2003). UV filters with antagonistic action at androgen receptors in the MDA-kb2 cell transcriptional-activation assay. Toxicol Sci.

[50] Böttcher M, Teigeler M, Schäfers C, Braunbeck T (2007) Effects of endocrine active substances on populations of the zebrafish (*Danio rerio*). In: Poster presentation at SETAC Europe 17th annual meeting, Porto, Portugal

[51] Segner H, Caroll K, Fenske M, Janssen CR, Maack G, Pascoe D, Schäfers C, Vandenbergh GF, Watts M, Wenzel A (2003). Identification of endocrine-disrupting effects in aquatic vertebrates and invertebrates: report from the European IDEA project. Ecotoxicol Environ Saf.

[52] Sohoni P, Tyler CR, Hurd K, Caunter J, Hetheridge M, Williams T, Woods C, Evans M, Toy R, Gargas M, Sumpter JP (2001). Reproductive effects of long-term exposure to bisphenol A in the fathead minnow (*Pimephales promelas*). Environ Sci Technol.

[53] Nakazawa H, Yamaguchi A, Inoue K, Yamazaki T, Kato K, Yoshimura Y, Makino T (2002). In vitro assay of hydrolysis and chlorohydroxy derivatives of bisphenol A diglycidyl ether for estrogenic activity. Food Chem Toxicol.

[54] Satoh K, Ohyama K, Aoki N, Iida M, Nagai F (2004). Study on anti-androgenic effects of bisphenol A diglycidyl ether (BADGE), bisphenol F diglycidyl ether (BFDGE) and their derivatives using cells stably transfected with human androgen receptor, AR-EcoScreen. Food Chem Toxicol.

[55] Seki M, Yokota H, Matsubara H, Maeda M, Tadokoro H, Kobayashi K (2003). Fish full life-cycle testing for the weak estrogen 4-*tert*-pentylphenol on medaka (*Oryzias latipes*). Environ Toxicol Chem.

[56] Matthews JB, Fertuck KC, Celius T, Huang YW, Fong CJ, Zacharewski TR (2002). Ability of structurally diverse natural products and synthetic chemicals to induce gene expression mediated by estrogen. J Steroid Biochem Mol Biol.

[57] Moffat GJ, Burns A, Van Miller J, Joiner R, Ashby J (2001). Glucuronidation of nonylphenol and octylphenol eliminates their ability to activate transcription via the estrogen receptor. Regul Toxicol Pharmacol.

[58] Yokota H, Seki M, Maeda M, Oshima Y, Tadokoro H, Honjo T, Kobayashi K (2001). Life-cycle toxicity of 4-nonylphenol to medaka (*Oryzias latipes*). Environ Toxicol Chem.

[59] Liu C, Du Y, Zhou B (2007). Evaluation of estrogenic activities and mechanism of action of perfluorinated chemicals determined by vitellogenin induction in primary cultured tilapia hepatocytes. Aquat Toxicol.

[60] Hill EM, Smith MD (2006). Identification and steroid receptor activity of products formed from the bromination of technical nonylphenol. Chemosphere.

[61] Kinnberg K, Holbech H, Petersen GI, Bjerregaard P (2007). Effects of the fungicide prochloraz on the sexual development of zebrafish (*Danio rerio*). Comp Biochem Physiol C Toxicol Pharmacol.

[62] Andersen HR, Vinggaard AM, Hoj Rasmussen T, Gjermandsen IM, Bonefeld-Jorgensen CE (2002). Effects of currently used pesticides in assays for estrogenicity, androgenicity, and aromatase activity in vitro. Toxicol Appl Pharmacol.

