# Supplementary material for: Screening for potential endocrine disruptors in fish: evidence from structural alerts and in vitro and in vivo toxicological assays
Source: Environ Sci Eur. 2016 Nov 2;28(1):26. doi: 10.1186/s12302-016-0094-5 (PMC5093190; doi:10.1186/s12302-016-0094-5)
Supplement: Supplementary file 1 — Additional file 1. Estrogenic and androgenic endocrine activities in vitro. [file 12302_2016_94_MOESM1_ESM.zip › SI_1 UBA endocrine Text.docx]

## SI1: Estrogenic and androgenic endocrine activities *in vitro*

**Supporting information** to:

Screening for potential endocrine disruptors in fish: evidence from structural alerts and *in vitro* and *in vivo* toxicological assays

Monika Nendza (nendza@al-luhnstedt.de), Analytical Laboratory, Bahnhofstr. 1, 24816 Luhnstedt, Germany

Andrea Wenzel (andrea.wenzel@ime.fraunhofer.de) and Martin Müller (martin.mueller@ime.fraunhofer.de), Fraunhofer Institute for Molecular Biology and Applied Ecology IME, Auf dem Aberg 1, 57392 Schmallenberg, Germany

Geertje Lewin (g.lewin@web.de) and Nelly Simetska (nelly.simetska@item.fraunhofer.de), Fraunhofer Institute for Toxicology and Experimental Medicine ITEM, Nikolai-Fuchs-Str. 1, 30625 Hannover, Germany

Frauke Stock (frauke.stock@uba.de) and Jürgen Arning (Juergen.Arning@uba.de), German Environment Agency UBA, Wörlitzer Platz 1, 06844 Dessau-Roßlau, Germany….

The following files provide a collection of data from receptor-based in vitro assays for the intrinsic potential of chemicals to interact with estrogen and androgen (EA) receptors:

- SI_1 DSSTOX.xlsx
  Data from National Center for Toxicological Research Estrogen Receptor Binding Database (DSSTOX-NCTRER): http://www.epa.gov/ncct/dsstox/sdf_nctrer.html)
- SI_1 FDA_AndrogenReceptorBinding.xlsx
  Data from U.S. Food and Drug Administration Endocrine Disruptor Knowledge Base (FDA-EDKB): http://www.fda.gov/ScienceResearch/BioinformaticsTools/EndocrineDisruptorKnowledgebase/default.htm)
- SI_1 Test_Results.xlsx
  Data collected from the literature
