# Supplementary material for: Screening for potential endocrine disruptors in fish: evidence from structural alerts and in vitro and in vivo toxicological assays
Source: Environ Sci Eur. 2016 Nov 2;28(1):26. doi: 10.1186/s12302-016-0094-5 (PMC5093190; doi:10.1186/s12302-016-0094-5)
Supplement: Supplementary file 2 — Additional file 2. Structural alerts. [file 12302_2016_94_MOESM2_ESM.docx]

## SI 2: Structural alerts

**Supplemental information** to:

Screening for potential endocrine disruptors in fish: evidence from structural alerts and *in vitro* and *in vivo* toxicological assays

Monika Nendza ([nendza@al-luhnstedt.de](mailto:nendza@al-luhnstedt.de)), Analytical Laboratory, Bahnhofstr. 1, 24816 Luhnstedt, Germany *

Andrea Wenzel ([andrea.wenzel@ime.fraunhofer.de](mailto:andrea.wenzel@ime.fraunhofer.de)) and Martin Müller ([martin.mueller@ime.fraunhofer.de](mailto:martin.mueller@ime.fraunhofer.de)), Fraunhofer Institute for Molecular Biology and Applied Ecology IME, Auf dem Aberg 1, 57392 Schmallenberg, Germany

Geertje Lewin ([g.lewin@web.de](mailto:g.lewin@web.de)) and Nelly Simetska ([nelly.simetska@item.fraunhofer.de](mailto:nelly.simetska@item.fraunhofer.de)), Fraunhofer Institute for Toxicology and Experimental Medicine ITEM, Nikolai-Fuchs-Str. 1, 30625 Hannover, Germany

Frauke Stock ([frauke.stock@uba.de](mailto:frauke.stock@uba.de)) and Jürgen Arning ([Juergen.Arning@uba.de](mailto:Juergen.Arning@uba.de)), German Environment Agency UBA, Wörlitzer Platz 1, 06844 Dessau-Roßlau, Germany

The following tables provide a collection of structural alerts from the literature to detect potential endocrine disruptors (EDs) (Table SI 2A: Chemical structures related to interactions with estrogen receptors, Table SI 2B: Chemical structures related to interactions with androgen receptors). Most currently known EDs operate via sexual endocrine pathways and estrogenic and androgenic EDs (EA-EDs) are thus an important group of possible SVHC candidates under REACH. The experimental data that were analysed to derive the structural alerts, cover sexual endocrine activities of substances, the effects of which are mediated by competitive interactions with EA receptors. Accordingly, the structural alerts derived with these data are limited to the identification of potential EA-EDs.

Notably, many chemicals are potential ligands of both, the estrogen and androgen receptors. Substantial similarity of EA receptors regarding their binding sites can thus be assumed. Only a few chemical classes interact only with one of these receptor families, such as phthalates [1].

**Table SI 2A: Chemical structures related to interactions with estrogen receptors. M_P_: Structure of the pharmacophore, M_S_: Substructure.**

| **Chemical structure** | **M_P_** | **M_S_** | **Structural alert (SA)** | **Description** | **Endpoint** | **Binding affinity** | **Data set** | **Comments** | **References** |
| --- | --- | --- | --- | --- | --- | --- | --- | --- | --- |
| **Steroids** |  |  |  |  |  |  |  |  |  |
|  | ⮟ |  | SA 01  steroid backbone with any pattern of double bonds | Steroid backbone (with aromatic A Ring) | ER binding | strong | Training set: n=232 (NCTR) | Steroid backbone of 17ß-estradiol | DSSTox [2] |
|  | ⮟ |  | SA 01  steroid backbone with any pattern of double bonds | Steroid backbone | ER binding | --- | Training set: n=232 (NCTR) | any pattern of bonds | Hong et al. [3] |
|  | ⮟ |  | SA 02 partial steroid structure without restrictions (see comments), conservative approach | Partial steroid structure | ER binding | --- | Diverse literature data  Statistics: r²=0.96  sensitivity:100%  specificity: NA | C1, C2: aromatic  C3: aromatic, aliphatic  C4: aliphatic | Klopman & Chakravati [4] |
|  | ⮟ |  | SA 03 partial steroid structure without restrictions (see comments), conservative approach | Partial steroid backbone | ER binding | --- | Diverse literature data | C1, C2: aromatic  C3: aromatic, aliphatic  C4: aliphatic | Klopman & Chakravati [5] |
| **DES** |  |  |  |  |  |  |  |  |  |
|  | ⮟ |  | SA 04  without restrictions (see comments), conservative approach | DES backbone | ER binding | strong | Training set: N=232 (NCTR) | DES has p-OH on both rings  R is not defined  Ethyl substitution of the ethenyl bridge allowed | DSSTox [2] |
|  | ⮟ |  | SA 05  without restrictions (see comments), conservative approach | DES backbone (hexestrol derivative) | ER binding | strong | Training set: N=232 (NCTR) | p-OH or p-OR on one ring  R is not defined  Methyl, ethyl substitution of the ethenyl bridge allowed | DSSTox [2] |
|  | ⮟ |  | SA 06 | DES backbone (including most phytoestrogens [6]) | ER binding | --- | Training set: N=232 (NCTR) | Any binding of substituents, bridge | Hong et al. [3] |
|  | ⮟ |  |  | 2 aromatic rings, connected by 2 C atoms  examples: steroids, biogenic estrogens, diphenylethanes | ER RBA % | log RBA %: +2,60 to -3,67  1 of 5 inactive | 188 diverse structures  (100 binding, 88 non-binding) | Stronger activity than compounds with only 1 ring or 2 rings connected by 1 C atom  Binding of agonists as well as antagonists | Blair et al. [1] |
|  | ⮟ |  | SA 07  without restrictions (see comments), conservative approach | DES backbone (triphenylethylen derivative) | ER binding | strong | Training set: N=232 (NCTR) | Antiestrogenes  Ethyl and alkoxyamine substitution allowed  4-OH substitution allowed | DSSTox [2] |
| **Phytoestrogenes** |  |  |  |  |  |  |  |  |  |
|  | ⮟ |  | SA 08  without restrictions (see comments), conservative approach | Phytoestrogenes  Flavones | ER binding | weak | Training set: N=232 (NCTR) | Includes DES backbone, OH substituents on one or more rings allowed | DSSTox [2] |
|  | ⮟ |  | SA 09  without restrictions (see comments), conservative approach | Phytoestrogenes  Flavones | ER binding | weak | Training set: N=232 (NCTR) | Includes DES backbone, OH substituents on one or more rings allowed | DSSTox [2] |
|  | ⮟ |  | SA 10  without restrictions (see comments), conservative approach | Phytoestrogenes  Isoflavones | ER binding | weak | Training set: N=232 (NCTR) | Includes DES backbone, one or more OH or OR substituents on one or more rings allowed | DSSTox [2] |
|  | ⮟ |  | SA 11  without restrictions (see comments), conservative approach | Phytoestrogenes  Coumestanes | ER binding | weak to moderate | Training set: N=232 (NCTR) | Includes DES backbone, at C4: ethyl substitution or O-bridge to C2’ allowed  at C4’: OH and OCH3 substitution allowed | DSSTox [2] |
|  | ⮟ |  | SA 12  without restrictions (see comments), conservative approach | Phytoestrogenes  Chalcanoides | ER binding | weak | Training set: N=232 (NCTR) | Very similar to flavanones  p-OH substitution at one or both rings allowed | DSSTox [2] |
|  | ⮟ |  | SA 13  without restrictions (see comments), conservative approach, double bond C11=C12 considered | Mycoestrogenes | ER binding | weak to moderate | Training set: N=232 (NCTR) | Double bond C11=C12 allowed  OH or Oxo substitution at C7 allowed | DSSTox [2] |
|  |  |  |  | Flavonoides | estrogenic gene expression  EC50 [mol/l] | 8 of 15 inactive | 120 aromatic compounds | --- | Schultz et al. [7] |
| **Diphenylmethanes** |  |  |  |  |  |  |  |  |  |
|  | ⮟ |  | SA 14 | 2 aromatic rings separated by 1 C atom  Examples: Bisphenol A derivatives, benzophenones | ER RBA % | log RBA %: +2,60 to -3,67  2 of 8 inactive | 188 diverse structures  (100 binding, 88 non-binding) | Less active than 2 aromatic rings separated by 2 C atoms  Binding of agonists as well as antagonists | Blair et al. [1] |
|  | ⮟ |  | SA 14  (conservative version) | Diphenylmethanes  Diphenolalkanes | ER binding | weak | Training set: N=232 (NCTR) | 3. ring at C bridge allowed  diverse substituents at C bridge and phenol rings allowed  4-OH required for binding  2,6-bulk reduces binding affinity | DSSTox [2] |
|  | ⮟ |  | SA 15 benzophenone with any substitution | Diphenylmethanes Benzophenones | ER binding | weak | Training set: N=232 (NCTR) | min. 1 phenolic ring  Carbonyl or sulfonyl bridge  diverse OH and OCH_3_ substitution allowed | DSSTox [2] |
|  | ⮟ |  |  | Benzophenones | ER RBA % | 3 of 5 inactive | 188 diverse structures | --- | Blair et al. [1] |
|  | ⮟ |  |  | Benzophenones | estrogenic gene expression  EC50 [mol/l] | 8 of 15 inactive | 120 aromatic compounds | --- | Schultz et al. [7] |
|  | ⮟ |  | SA 16  without restrictions (see comments), conservative approach | Diphenylmethanes  DDTs | ER binding | weak | Training set: N=232 (NCTR) | CClx (x=2 or 3) with single or double bond to C bridge  diverse OH, OCH3 and Cl substitution allowed | DSSTox [2] |
|  | ⮟ |  | SA 17  any substitution allowed, conservative approach | Methoxychlor analoga | ER RBA % | 2 of 7 inactive | 188 diverse structures | -X: =CCl2, -CCl3  R1, R2: H, OH, OMe | Blair et al. [1] |
|  | ⮟ |  | SA 17 | DDT analogs and bisphenols | ER binding | --- | Training set: N=232 (NCTR) | OH, Cl substitution at aromatic rings allowed  Alkyl, Cl substitution at bridge allowed | Shi et al. [6] |
| **Biphenyls** |  |  |  |  |  |  |  |  |  |
|  | ⮟ |  | SA 18 | PCBs | Antiestrogenic in MCF-7 cells | --- | Cl6, Cl5 and Cl4 PCBs, div. Aroclors | koplanar PCBs are more active, mono-ortho-koplanar PCBs are active only at high concentrations  Aroclors are inactive | Krishnan & Safe [8] |
|  | ⮟ |  |  | PCBs | competitive ER binding | --- | 4 OH PCBs | non-koplanar PCBs (= ortho substitution) are estrogenic | Waller et al. [9] |
|  | ⮟ |  |  | PCBs | competitive ER binding | --- | 4 OH PCBs | ↑ binding affinity with:  ↑ size and ↓ branching  ↑ HOMO-LUMO-Gap | Bradbury et al. [10] |
|  | ⮟ |  |  | PBBs | DR-CALUX | --- | 2 PCBs  1 PBB | polybrominated biphenyls can bind to AhR, probably in non-coplanar conformation | Navas et al. [11] |
|  | ⮟ |  |  | PBBs | ER RBA % | 3 of 9 inactive | 188 diverse structures | --- | Blair et al. [1] |
|  | ⮟ |  |  | Biphenyls | ER RBA % | 1 of 3 inactive | 188 diverse structures | Active: 4-phenylphenol, 3-phenylphenol  Inactive: 2-phenylphenol | Blair et al. [1] |
|  | ⮟ |  |  | Biphenyls | estrogenic gene expression  EC50 [mol/l] | 3 of 21 inactive | 120 aromatic compounds | --- | Schultz et al. [7] |
|  | ⮟ |  | SA 19  with or without OH substitution (conservative approach) | Biphenyls (polychlorinated) | ER binding | weak | Training set: N=232 (NCTR) | ≥1 Cl substituent  OH substitution allowed | DSSTox [2] |
|  | ⮟ |  | SA 20 | Biphenyls  (not chlorinated) | ER binding | weak | Training set: N=232 (NCTR) | min. 1 phenolic ring | DSSTox [2] |
| **Dioxins, Furans** |  |  |  |  |  |  |  |  |  |
|  | ⮟ |  | SA 21 | Dibenzodioxins | Antiestrogenic in MCF-7 cells | --- | Cl_5_ dibenzodioxins | Most activity with 4 lateral Cl (position 2,3,7,8) | Krishnan & Safe [8] |
|  | ⮟ |  | SA 22 | Dibenzofurans | Antiestrogenic in MCF-7 cells | --- | Cl4 and Cl5 dibenzofurans | Most activity with 4 lateral Cl (position 2,3,7,8) | Krishnan & Safe [8] |
| **Phthalates** |  |  |  |  |  |  |  |  |  |
|  | ⮟ |  | SA 23 | Phthalates | ER RBA % | 8 of 8 inactive | 188 diverse structures | Phthalates bind to AR, but not to ER! | Blair et al. [1] |
| **Phenols** |  |  |  |  |  |  |  |  |  |
|  | ⮟ | ⮟ | all phenols are covered by SA 26 | Alkylphenols | ER binding | very weak | Training set: N=232 (NCTR) | diverse Cl or alkyl substitution allowed  ↑ binding affinity with ↑ log *K*_OW_ (para-substituted), maximum at C9 | DSSTox [2] |
|  | ⮟ | ⮟ |  | Alkylphenols | ER RBA % | 3 of 21 inactive | 188 diverse structures | diverse Cl or alkyl substitution  inactive: 2-ethylphenol, eugenol, isoeugenol | Blair et al. [1] |
|  | ⮟ | ⮟ | SA 24 | Parabens | ER binding | very weak | Training set: N=232 (NCTR) | para-substitution required  ↑ binding affinity with ↑ log *K*_OW_ | DSSTox [2] |
|  | ⮟ | ⮟ |  | Parabens | ER RBA % | 0 von 7  inactive | 188 diverse structures | --- | Blair et al. [1] |
|  | ⮟ | ⮟ | SA 25 | Alkoxyphenols | ER binding | very weak | Training set: N=232 (NCTR) | diverse alkoxy substitution | DSSTox [2] |
|  |  | ⮟ |  | Phenols | --- | --- | --- | No ortho substitution,  meta- para-: bulky, hydrophobic substitution | Katzenellenbogen [12] |
|  |  | ⮟ |  | Phenols | ER binding | --- | Training set: N=232 (NCTR) | Not specified | Hong et al. [3] |
|  |  | ⮟ |  | Phenols | ER RBA % | --- | 188 diverse structures | ↑ binding affinity with  ↑ chain length (maximum at ~ C8) | Blair et al. [1] |
|  |  | ⮟ |  | Phenols | estrogenic gene expression  EC50 [mol/l] | --- | 29 Alkylphenols | ↑ binding affinity with:  ↑ chain length (≥3, max. 8)  ↑ branching  ↑ ortho → meta → para | Routledge & Sumpter [13] |
|  |  | ⮟ |  | Phenols | estrogenic gene expression  EC50 [mol/l] | --- | 29 Alkylphenols from [13] | Decision trees:  Lmax (~chain length)  Distance O_C(sp³) (~branching)  LUMO (~preferred para-substitution) | Schmieder et al. [14] |
|  |  | ⮟ |  | Phenols | estrogenic gene expression  EC50 [mol/l] | --- | 120 aromatic compounds | p-substitution: hydrophobic, >3 atoms | Schultz et al. [7] |
|  |  | ⮟ |  | Phenols | ER binding | --- | Diverse literature data  Statistics:  r²=0,90  Sensitivity 99%  Specificity 77% | No ortho-substitution, max. 1 meta-substituent, para-substituent allowed  alternative: 1 or 2 OH or Cl in ortho position  Modulators: ↑ binding affinity with: ↑ *K*_OW_, 2. OH at 12,8 Å distance | Klopman & Chakravati [4] |
|  |  | ⮟ |  | Phenols | ER binding | --- | Diverse literature data | No ortho-substitution, max. 1 meta-substituent, para-substituent allowed  alternative: 2 meta substituents and/or 1 para-substituent | Klopman & Chakravati [5] |
|  |  | ⮟ |  | Phenols | Diverse | --- | Diverse literature data | No ortho-Alkyl substitution | Jordan et al. [15] |
| **Diverse structures** |  |  |  |  |  |  |  |  |  |
| --- |  |  |  | Pesticides | ER RBA % | 18 von 19  inactive | 188 diverse structures | Active: kepone  Inactive: 2,4,5-T, 2,4-D, α-chlordane, alachlor, aldrin, atrazin, carbaryl, carbofuran, dieldrin, endosulfan, heptachlor, hexachlorbenzene, lindane, metolachlor, mirex, prometon, simazin, vinclozolin | Blair et al. [1] |
|  |  |  |  | “miscellaneous compounds“ | ER RBA % | --- | 188 diverse structures | Inactive:  All tested  - acids (N=4)  - alcohols (N=4)  - aldehydes (N=3)  - amines (N=5)  - hydrocarbons (N=6)  Active:  - 2 of 5 ethers/esters  (4-heptyloxyphenol, 4-benzyloxyphenol)  - 4 of 11 “others“  (aurin, nordihydroguaiaretic acid, phenol red, phenolphthalein) | Blair et al. [1] |
| **Other substructures** |  |  |  |  |  |  |  |  |  |
|  |  |  | SA 27 | Ketonic carbonyl | ER binding | --- | Diverse literature data | No ortho-substitution, max. 1 meta substituent, para-substitution allowed | Klopman & Chakravati [4] |
|  |  | ⮟ | NOT used as structural alert, see comment | Single aromatic ring | ER RBA % | log RBA %: +2,60 to -3,67 | 188 diverse structures  (100 binding, 88 non-binding) | Less active than 2 aromatic rings separated by 2 C atoms  Examples: alkylphenols, phthalates, parabens  Binding of agonists as well as antagonists | Blair et al. [1] |
|  |  | ⮟ |  | Ring structure | estrogenic gene expression  EC50 [mol/l] | --- | 120 aromatic compounds | Rings without OH are inactive | Schultz et al. [7] |

**Table SI 2B: Chemical structures related to interactions with androgen receptors. M_P_: Structure of the pharmacophore, M_S_: Substructure.**

| **Chemical structure** | **M_P_** | **M_S_** | **Structural alert (SA)** | **Description** | **Endpoint** | **Binding affinity** | **Data set** | **Comments** | **References** |
| --- | --- | --- | --- | --- | --- | --- | --- | --- | --- |
| **Steroids** |  |  |  |  |  |  |  |  |  |
|  | ⮟ |  | SA 01 | Steroid backbone | AR binding | --- | 202 diverse structures | Androgenic and estrogenic steroids bind to AR and ER  difference:  3-Keto at A ring: more likely AR binding  3-OH at A ring: more likely ER binding | Fang et al. [16] |
| **DES** |  |  |  |  |  |  |  |  |  |
|  | ⮟ |  | SA 05 | DES backbone | AR binding | --- | 202 diverse structures | DES analogues bind to AR and ER  difference:  3-Keto at A ring: more likely AR binding  3-OH at A ring: more likely ER binding | Fang et al. [16] |
| **Phytoestrogens** |  |  |  |  |  |  |  |  |  |
| Structures see Table SI1A | ⮟ |  |  | Flavones  Flavanones  Isoflavones  Coumestanes  Chalcanoides  Mycoestrogenes | AR binding | 3 of 17  inactive | 202 diverse structures | ↑ Binding affinity with ↑ log *K*_OW_ Non-binder have very low log *K*_OW_  Inactive: coumestrol, 7-hydroxyflavone, naringin | Fang et al. [16] |
| **Diphenylmethanes** |  |  |  |  |  |  |  |  |  |
|  | ⮟ |  | SA 17 | Methoxychlor analoga | AR binding | 0 of 5  inactive | 202 diverse structures | -X: =CCl_2_, -CCl_3_  R1, R2: OH, OMe | Fang et al. [16] |
|  | ⮟ |  | SA 15 | Benzophenones | AR binding | 0 of 4  inactive | 202 diverse structures | R: H, OH | Fang et al. [16] |
|  | ⮟ |  | SA 15 | Benzophenones | AR binding | 0 of 4  inactive  2 of 4:  agonists | N=66 (SPEED 98) | X: O, CH_2_  R1: H, Cl, OH | Tamura et al. [17] |
|  | ⮟ |  | SA 17 | DDT analoga | AR binding | 0 of 6  inactive | 202 diverse structures | X: =CCl_2_, -CHCl_2_, -CCl_3_  2. ring: Cl in o- or p- | Fang et al. [16] |
|  | ⮟ |  | SA 17 | Bisphenols | AR binding | 0 of 3  inactive | 202 diverse structures | R1: methyl or ethyl  R2: H, OH | Fang et al. [16] |
|  | ⮟ |  | SA 17 | DDT analoga and Bisphenols | AR binding | 0 of 11  inactive | N=66 (SPEED 98) | R1: H, OH, CH_3_  R2: CCl_2_, CCl_3_, C(O)O-alkyl, 3-pyrimidine, CH_3_  R3: Cl, Br, OCH_3_, OH  R4: H, Cl, Br, OCH_3_, OH  R5: H, Cl | Tamura et al. [17] |
|  | ⮟ |  | SA 16 | DDE analoga | AR binding | 0 of 2  inactive | N=66 (SPEED 98) | R1: H, Cl  R2: H, Cl | Tamura et al. [17] |
|  |  | ⮟ | SA 28 | Triphenylmethanes | AR binding | 2 of 3  inactive | 202 diverse structures | Active: aurin  Inactive: phenol red, phenolphthalein | Fang et al. [16] |
|  | ⮟ |  | SA 29 | Sulfonyldiphenols | AR binding | 0 of 1  inactive | 202 diverse structures |  | Fang et al. [16] |
| **Diphenylether** |  |  |  |  |  |  |  |  |  |
|  | ⮟ |  | SA 30 | Diphenylether | AR binding | 0 of 5  inactive  3 of 5:  agonists | N=66 (SPEED 98) | R1: Cl, NO_2_  R2: H, CH_3_O, C(O)OCH_3_  R3: H, Cl  R4: H, Cl | Tamura et al. [17] |
| **Biphenyls** |  |  |  |  |  |  |  |  |  |
|  | ⮟ |  | SA 18 | PCBs | AR binding | 1 of 6  inactive | 202 diverse structures | Substituents : H, OH, Cl | Fang et al. [16] |
| **Isoindoles** |  |  |  |  |  |  |  |  |  |
|  | ⮟ |  | SA 31 | Isoindoles | AR binding | 0 of 3  inactive | 202 diverse structures | R: H, NO_2_, OH | Fang et al. [16] |
| **Hydrocarbons (aromatic)** |  |  |  |  |  |  |  |  |  |
| --- | ⮟ |  |  | Aromatic hydrocarbons | AR binding | 6 of 7  inactive | 202 diverse structures | Active: triphenylethylene  Inactive: sec-butylbenzene, n-butylbenzene, 1,6-dimethylnaphthalene, diphenylbutadiene, chrysene, triphenylpropane | Fang et al. [16] |
| --- | ⮟ |  |  | PAK | AR binding | 1 of 7  inactive  4 of 6:  agonists | N=66 (SPEED 98) | Active (agonist): benz[a]pyrene, perylene, chrysene, phenanthrene  Active (antagonist): pyrene, antracene  Inactive: naphtalene | Tamura et al. [17] |
| **Phthalates** |  |  |  |  |  |  |  |  |  |
|  | ⮟ |  | SA 23 | Phthalate ester | AR binding | 1 of 7  inactive | 202 diverse structures | R1, R2: alkyl  Inactive: bis(2-ethylhexyl)-phthalate | Fang et al. [16] |
|  | ⮟ |  |  | Phthalate ester | AR binding | 0 of 5 inactive | N=66 (SPEED 98) | AR antagonists  R1, R2: alkyl | Tamura et al. [17] |
| **Phenols** |  |  |  |  |  |  |  |  |  |
|  | ⮟ |  | SA 26 | Alkylphenols, Parabens, Alkoxyphenols | AR binding | 5 of 19  inactive | 202 diverse structures | ↑ Binding affinity with ↑ log *K*_OW_  Non-binder have very low log *K*_OW_  Inactive: vanillin, phenol, methylparaben, 2-chlorophenol, 4-ethylphenol | Fang et al. [16] |
|  | ⮟ |  | SA 26 | Alkylphenols | AR binding | 1 of 7  inactive | N=66 (SPEED 98) | p-Alkylphenols (pentyl- to nonyl); 2,4-Cl_2_-phenol: very low binding affinity | Tamura et al. [17] |
|  |  | ⮟ | SA 26 | Phenol analoga | AR binding | 2 of 6  inactive | 202 diverse structures | Active: 1-methoxy-4-[1-propylphenyl] benzene, carbaryl, nordihydroguaiaretic acid, 4-(3,5-diphenylcyclohexyl)phenol,  Inactive: 2,6-dihydroxyanthrachinone, 2-naphthol | Fang et al. [16] |
|  |  | ⮟ |  | Aromatic phosphoric acid ester | AR binding | 0 of 3  inactive | 202 diverse structures | Active: methylparathion, ethylparathion, triphenylphosphate | Fang et al. [16] |
| **Flutamides** |  |  |  |  |  |  |  |  |  |
|  | ⮟ |  | SA 36 | Flutamides | AR binding | 1 of 9  inactive | 202 diverse structures | ↑ Binding affinity with ↑ e-affinity of substituents at ring  Non-binder have e-donating substituents at ring  Inactive: p-lactophenetid | Fang et al. [16] |
|  |  | ⮟ | SA 36 | “miscellaneous compounds“ | AR binding | 1 of 8  inactive  2 of 7:  agonists | N=66 (SPEED 98) | 7 of the 8 compounds have a common substructure (with X_1_: N, O, C, X_2_=X_3_: C=O, P=S, at ring: 1 to 2 m-, p-substituents): flutamide, linuron, fenitrothion, ethyl-parathion, vinclozoline, procymidon, iprodion (inactive), Exception with a different structure: octachlorostylene (active) | Tamura et al. [17] |
| **Aromatic acids** |  |  |  |  |  |  |  |  |  |
|  |  | ⮟ |  | Aromatic acids, amides, esters | AR binding | 4 of 6  inactive | 202 diverse structures | X: O, N  Ring substituents: NH_2_, OH, O-alkyl  Active: 4-aminobutylbenzoate, 4-heptyloxybenzoic acid | Fang et al. [16] |
| **Triazines** |  |  |  |  |  |  |  |  |  |
|  |  | ⮟ | SA 33 | Triazines | AR binding | 3 of 3  inactive | 202 diverse structures | Inactive: simazine, atrazine, prometone | Fang et al. [16] |
| **Diverse structures** |  |  |  |  |  |  |  |  |  |
| --- |  |  |  | Organochlorines | AR binding | 3 of 10  inactive | 202 diverse structures | Active: 2,4,5-T, γ-hexachlorocyclohexane, aldrine, endosulfane, heptachlor, kepone, chlordane  Inactive: 2,4-D, hexachlorobenzene, mirex | Fang et al. [16] |
| --- |  |  |  | Organochlorines | AR binding | 11 of 17  inactive | N=66 (SPEED 98) | Active: dieldrine, endrine, aldrine, toxaphene, γ-hexachlorocyclohexane, trans-heptachlorepoxid  Inactive: heptachlor, trans-nonachlor, chlordane, trans-chlordane, cis-chlordane, oxychlordane, chlordecone, mirex, ß-hexachlorocyclohexane, hexachlorbenzene, cis-heptachlorepoxid | Tamura et al. [17] |
| --- |  |  |  | Non-cyclic compounds | AR binding | 8 of 10  inactive | 202 diverse structures | Active: diisobutyladipate, dibutyladipate | Fang et al. [16] |
| --- |  |  |  | Siloxanes | AR binding | 1 of 3  inactive | 202 diverse structures | Active: 1,3-diphenyltetramethyldisiloxane, triphenylsilanole  Inactive: 1,3-dibenzyltetramethyldisiloxane | Fang et al. [16] |

# References

1. Blair RM, Fang H, Branham WS, Hass BS, Dial SL, Moland CL, Tong W, Shi L, Perkins R, Sheehan DM (2000) The estrogen receptor relative binding affinities of 188 natural and xenochemicals: Structural diversity of ligands. Toxicol Sci 54:138-153

2. US EPA (2016) Distributed Structure-Searchable Toxicity (DSSTox) Database. https://www.epa.gov/chemical-research/distributed-structure-searchable-toxicity-dsstox-database

3. Hong H, Tong W, Fang H, Shi L, Xie Q, Wu J, Perkins R, Walker JD, Branham W, Sheehan DM (2002) Prediction of estrogen receptor binding for 58.000 chemicals using an integrated system of a tree-based model with structural alerts. Environ Health Persp 110:29-36

4. Klopman G, Chakravarti SK (2003) Structure-activity relationship study of a diverse set of estrogen receptor ligands (I) using MultiCASE expert system. Chemosphere 51:445-459

5. Klopman G, Chakravarti SK (2003) Screening of high production volume chemicals for estrogen receptor binding activity (II) by the MultiCASE expert system. Chemosphere 51:461-468

6. Shi L, Tong W, Fang H, Tong W, Wu J, Perkins R, Blair RM, Branham WS, Dial SL, Moland CL, Sheehan DM (2001) QSAR models using a large diverse set of estrogens. J Chem Inf Comput Sci 41:186-195

7. Schultz TW, Sinks GD, Cronin MTD (2002) Structure-activity relationships for gene activation oestrogenicity: evaluation of a diverse data set of aromatic chemicals. Environ Toxicol 17:14-23

8. Krishnan V, Safe S (1993) Polychlorinated Biphenyls (PCBs), Dibenzo-p-dioxins (PCDDs), and Dibenzofurans (PCDFs) as Antiestrogens in MCF-7 Human Breast Cancer Cells: Quantitative Structure-Activity Relationships. Toxicol Appl Pharmacol 120:55-61

9. Waller CL, Minor DL, McKinney JD (1995) Using Three-Dimensional Quantitative Structure-Activity Relationships to Examine Estrogen Receptor Binding Affinities of Polychlorinated Hydroxybiphenyls. Environ Health Persp 103:702-707

10. Bradbury SP, Mekenyan OG, Ankley GT (1996) Quantitative structure-activity relationships for polychlorinated hydroxybiphenyl estrogen receptor binding affinity: an assessment of conformer flexibility. Environ Toxicol Chem 15:1945-1954

11. Navas JM, Alonso M, Casado S, Miranda C, Tarazona JV, Herradón B (2007) Structural features of ligands of the aryl hydrocarbon receptor: The case of the polybrominated biphenyl 209 (PBB-209). Poster Presentation at SETAC Europe 17th Annual Meeting, Porto, Portugal

12. Katzenellenbogen JA (1995) The structural pervasiveness of estrogenic activity. Env Health Persp 103:99-101

13. Routledge EJ, Sumpter JP (1997) Structural features of alkylphenolic chemicals associated with estrogenic activity. J Biol Chem 272:3280-3288

14. Schmieder P, Aptula AO, Routledge EJ, Sumpter JP, Mekenyan OG (2000) Estrogenicity of alkylphenolic compounds: A 3-D structure-activity evaluation of gene activation. Environ Toxicol Chem 19:1727-1740

15. Jordan VC, Mittal S, Gosden B, Koch R, Lieberman ME (1985) Structure-activity relationships of estrogens. Environ Health Persp 61:97-110

16. Fang H, Tong W, Branham WS, Moland CL, Dial SL, Hong H, Xie Q, Perkins R, Owens W, Sheehan DM (2003) Study of 220 natural, synthetic, and environmental chemicals for binding to the androgen receptor. Chem Res Toxicol 16:1338-1358

17. Tamura H, Ishimoto Y, Fujikawa T, Aoyama H, Yoshikawa H, Akamatsu M (2006) Structural basis for androgen receptor agonists and antagonists: Interaction of SPEED 98-listed chemicals and related compounds with the androgen receptor based on an in vitro reporter gene assay and 3 D-QSAR. Bioorg Med Chem 14:7160-7174
