# Supplementary material for: Screening for potential endocrine disruptors in fish: evidence from structural alerts and in vitro and in vivo toxicological assays
Source: Environ Sci Eur. 2016 Nov 2;28(1):26. doi: 10.1186/s12302-016-0094-5 (PMC5093190; doi:10.1186/s12302-016-0094-5)
Supplement: Supplementary file 3 — Additional file 3. False negatives (not recognized by structural alerts). [file 12302_2016_94_MOESM3_ESM.docx]

## SI 3: False negatives (not recognized by structural alerts)

**Supplemental information** to:

Screening for potential endocrine disruptors in fish: evidence from structural alerts and *in vitro* and *in vivo* toxicological assays

Monika Nendza ([nendza@al-luhnstedt.de](mailto:nendza@al-luhnstedt.de)), Analytical Laboratory, Bahnhofstr. 1, 24816 Luhnstedt, Germany *

Andrea Wenzel ([andrea.wenzel@ime.fraunhofer.de](mailto:andrea.wenzel@ime.fraunhofer.de)) and Martin Müller ([martin.mueller@ime.fraunhofer.de](mailto:martin.mueller@ime.fraunhofer.de)), Fraunhofer Institute for Molecular Biology and Applied Ecology IME, Auf dem Aberg 1, 57392 Schmallenberg, Germany

Geertje Lewin ([g.lewin@web.de](mailto:g.lewin@web.de)) and Nelly Simetska ([nelly.simetska@item.fraunhofer.de](mailto:nelly.simetska@item.fraunhofer.de)), Fraunhofer Institute for Toxicology and Experimental Medicine ITEM, Nikolai-Fuchs-Str. 1, 30625 Hannover, Germany

Frauke Stock ([frauke.stock@uba.de](mailto:frauke.stock@uba.de)) and Jürgen Arning ([Juergen.Arning@uba.de](mailto:Juergen.Arning@uba.de)), German Environment Agency UBA, Wörlitzer Platz 1, 06844 Dessau-Roßlau, Germany

Structural alerts can identify priority pollutants to undergo further assessments of their potential for endocrine disruption (ED). Screening based on structural alerts for estrogen and androgen (EA) receptor-based activities may ignore substances with other endocrine modes of action (danger of false negatives). The following table provides a detailed listing of chemicals with EA receptor-mediated endocrine activities *in vitro*, but not recognized by the structural alerts used in this study (see table 2 of this paper).

Closer inspection of the 50 chemicals with false negative predictions of EA-ED activities reveal 16 compounds with only (very) weak activities according to the *in vitro* test results. With regard to prioritisation of hazardous substances, these compounds may be considered less relevant. More significant are outliers with moderate (n=26), high (n=6) and very high (n=2) activity. Notably, among the 8 (very) highly active compounds are 6 antiandrogens like cypermethrin and linuron. The 26 false negative outliers of moderate activity are chemically diverse including several pharmaceuticals and pesticides. The search for relevant structural alerts to detect these compounds was not successful. Another influential factor may be related to uncertainties of individual test results.

**Table SI 3: Chemicals with EA endocrine activities in vitro, but not recognized by structural alerts (“false negatives“).**

FDA androgen, FDA estrogen: Receptor-ligand binding studies of androgenic and estrogenic compounds reported in the Endocrine Disruptor Knowledge Base from FDA (EDKB-FDA): <http://www.fda.gov/ScienceResearch/BioinformaticsTools/EndocrineDisruptorKnowledgebase/default.htm>

Androgen, Anti-androgen, Estrogen, Anti-estrogen: cell based test systems, for references of the individual substances refer to “SI_1 Test_Results.xlsx”

Receptor binding AR, Receptor binding ER: Receptor-ligand binding studies, for references of the individual substances refer to “SI_1 Test_Results.xlsx”

(1 and 2 = (very) high activity (marked in red), 3 = moderate activity (marked in yellow), 4 = weak activity, 5 = very weak or no activity).

|  | Ligand binding studies | | Cell based test systems | | | | | Ligand binding  studies | | | |  | |
| --- | --- | --- | --- | --- | --- | --- | --- | --- | --- | --- | --- | --- | --- |
| Name | FDA  androgen | FDA  estrogen | Androgen | Anti-  androgen | Estrogen | Anti-  estrogen | | Receptor  binding AR | | Receptor  binding ER | | Highest  classification | |
| 4:2 FTOH (1-Hexanol, 3,3,4,4,5,5,6,6,6-nonafluoro-; fluorotelomer alcohol) |  |  |  |  | 5 | 1 |  | |  | | 1 | |  |
| 2,4,6-Tribromophenol |  |  | 5 | 1 | 5 | 4 |  | |  | | 1 | |  |
| Homosalate (3,3,5-Trimethylcyclohexyl salicylate, HMS) |  |  | 5 | 2 | 5 | 5 |  | |  | | 2 | |  |
| Coumestrol | 5 | 3 |  |  | 2 |  |  | |  | | 2 | |  |
| 2-Hydroxy-4-methoxybenzophenone (Benzophenone-3, Bp-3) |  | 5 | 5 | 2 | 3 | 5 | 5 | | 5 | | 2 | |  |
| 1,1,4,4-Tetramethyl-6-ethyl-7-acetyl-1,2,3,4-tetrahydronaphthalene |  |  | 5 | 2 | 5 | 5 |  | |  | | 2 | |  |
| Cypermethrin |  |  | 5 | 2 | 5 | 3 |  | | 5 | | 2 | |  |
| Linuron | 4 |  |  | 2 |  |  |  | |  | | 2 | |  |
| 3-Hydroxybenzo[b]phenanthro[2,3-d]thiophene  (3-OH-B[b]PH[2,3-d]T) |  |  |  |  | 5 |  |  | | 3 | | 3 | |  |
| Deltamethrin |  |  |  | 5 | 5 | 3 |  | | 5 | | 3 | |  |
| Fenvalerate |  |  | 5 | 3 | 5 | 3 |  | | 5 | | 3 | |  |
| 3-Phenoxybenzoic acid (3-PBA) |  |  | 5 | 3 |  |  |  | |  | | 3 | |  |
| PFOA, Perfluorooctanoic acid |  |  |  |  | 3 | 5 |  | |  | | 3 | |  |
| Octachlorostyrene |  |  |  | 3 |  |  |  | |  | | 3 | |  |
| 1,1'-Diphenylethene |  |  |  | 3 |  |  |  | |  | | 3 | |  |
| Bis(2-hydroxyphenyl)methane (Bisphenol F) |  | 5 | 5 | 3 | 5 |  | 5 | | 5 | | 3 | |  |
| 2,4-Dichlorophenol |  |  |  | 3 |  |  |  | |  | | 3 | |  |
| 4-Diethylaminobenzaldehyde |  |  |  | 5 |  |  | 3 | |  | | 3 | |  |
| Anthracene |  |  | 5 | 3 |  |  |  | |  | | 3 | |  |
| Benzophenone | 4 |  |  | 3 |  |  |  | |  | | 3 | |  |
| Vinclozolin M1 |  |  |  | 3 |  |  |  | |  | | 3 | |  |
| Sumithrin |  |  |  |  | 4 | 3 |  | | 5 | | 3 | |  |
| Tetramethrin |  |  |  |  |  | 3 |  | | 5 | | 3 | |  |
| 3-Hydroxybenzo[b]naphtho[2,1-d]thiophene,  3-OH-B[b]N[2,1-d]T |  |  |  |  | 3 |  |  | | 3 | | 3 | |  |
| a,a-Dimethyl-b-ethyl allenolic acid |  |  |  |  |  |  |  | | 3 | | 3 | |  |
| Permethrin |  |  | 5 | 3 | 5 | 3 |  | | 5 | | 3 | |  |
| Vinclozolin M2 |  |  | 4 | 3 |  |  |  | |  | | 3 | |  |
| Neburon |  |  |  | 3 |  |  |  | |  | | 3 | |  |
| 4-tert-Butyl-4’-methoxy-dibenzoylmethan |  |  | 5 | 3 | 5 | 5 |  | | 5 | | 3 | |  |
| 6:2 FTOH, Perfluoroctanol |  |  |  |  | 3 | 5 |  | |  | | 3 | |  |
| trans-Chlorden (beta Chlordene) |  |  |  | 3 |  |  |  | |  | | 3 | |  |
| Allethrin (Bioallethrin) |  |  |  |  | 5 | 3 |  | | 5 | | 3 | |  |
| Lindane | 4 | 5 |  | 3 |  |  |  | | 5 | | 3 | |  |
| 2-tert-Butylanthraquinone |  |  | 3 |  |  |  | 5 | |  | | 3 | |  |
| Phenol, 4-chloro-2-methyl- | 4 | 5 |  |  |  |  |  | |  | | 4 | |  |
| 3,4-Diphenyltetrahydrofuran | 4 |  |  |  |  |  |  | |  | | 4 | |  |
| 1-Hydroxy-2-methoxy-4-propen-1-yl benzene (Isoeugenol, mixture of cis and trans) | 4 | 5 |  |  |  |  |  | | 5 | | 4 | |  |
| 2,2'-Methylenebis(4-chlorophenol) |  | 4 |  |  |  |  |  | | 5 | | 4 | |  |
| n-Butyl p-aminobenzoate | 4 | 5 |  |  |  |  |  | | 5 | | 4 | |  |
| 4,4'-Dichlorobenzophenone |  |  |  | 4 |  |  |  | |  | | 4 | |  |
| di-i-Butyl adipate | 4 |  |  |  |  |  |  | |  | | 4 | |  |
| Isoprothiolane |  |  |  |  | 4 |  |  | |  | | 4 | |  |
| p-Heptyloxybenzoic acid | 4 |  |  |  |  |  |  | |  | | 4 | |  |
| Dibutyl adipate | 4 |  |  |  |  |  |  | |  | | 4 | |  |
| Aurin | 4 | 4 |  |  |  |  |  | | 5 | | 4 | |  |
| Fenpicionil | 4 |  |  |  |  |  |  | |  | | 4 | |  |
| Propanil | 4 |  |  |  |  |  |  | |  | | 4 | |  |
| Metolachlor | 4 | 5 |  |  |  |  |  | | 5 | | 4 | |  |
| Flutolanil |  |  |  |  | 4 |  |  | |  | | 4 | |  |
| 2-sec-Butylphenol | 4 | 5 |  |  |  |  |  | | 5 | | 4 | |  |
